# Effects of Ethanol and Opioid Receptor Antagonists Naltrexone and LY2444296 on the Organization of Cholesterol- and Sphingomyelin-Enriched Plasma Membrane Domains

**DOI:** 10.1021/acschemneuro.5c00596

**Published:** 2025-09-26

**Authors:** Sho Oasa, Wai H. Mak, Adam L. Maddox, Carinna Lima, Andras Saftics, Lars Terenius, Tijana Jovanović-Talisman, Vladana Vukojević

**Affiliations:** 1 Center of Molecular Medicine, Department of Clinical Neuroscience, 27106 Karolinska Institutet, Stockholm 17176, Sweden; 2 Department of Cancer Biology & Molecular Medicine, Beckman Research Institute, 2523 City of Hope Comprehensive Cancer Center, Duarte, California 91010, United States

**Keywords:** ethanol, naltrexone, LY2444296, fluorescence correlation spectroscopy (FCS), single-molecule localization microscopy (SMLM), cholesterol/sphingomyelin domains

## Abstract

Excessive consumption of alcohol (ethanol (EtOH)) can lead to alcohol use disorder (AUD). While AUD can be managed through behavioral interventions, there is a great need for pharmacological remedies for this condition. To date, only three pharmacotherapeutic treatments for AUD have been approved by both the U.S. Food and Drug Administration and the European Medicines Agency. This is partly due to an incomplete understanding of the intricate molecular mechanisms through which EtOH affects cellular functions. Here, we focus on EtOH effects on nanoscale organization and the lateral dynamics of molecules in the plasma membrane, the primary cellular component that is affected by this compound. In a cell culture model natively expressing opioid receptors, important targets for medications aimed at preventing relapses in AUD, we used methods with single-molecule sensitivity to characterize the lateral organization and dynamics of cholesterol (Chol)- and sphingomyelin (SM)-enriched domains. Our data reveal that EtOH triggered a reorganization of Chol/SM-enriched domains and increased the plasma membrane fluidity. The general opioid receptor antagonist naltrexone (NTX), approved for relapse prevention in AUD, caused reorganization of Chol/SM-enriched domains, while pretreatment with NTX warded off EtOH-induced plasma membrane reorganization. In contrast, the selective kappa-opioid receptor antagonist LY2444296, at concentrations tested, showed a modest effect on Chol/SM-enriched domain organization and did not protect against EtOH-induced changes in plasma membrane organization. While the significance of these findings for AUD treatment remains uncertain at this stage, our study reveals that the action of NTX is not only limited to blocking opioid receptor activity by preventing agonist binding to the orthosteric binding site, but also is protective against short- and long-range EtOH-induced plasma membrane reorganization. By protecting against EtOH-induced changes in the lateral organization and dynamics of lipids in the plasma membrane, NTX may affect physiological outcomes through previously unrecognized noncanonical mechanisms.

## Graphical abstract

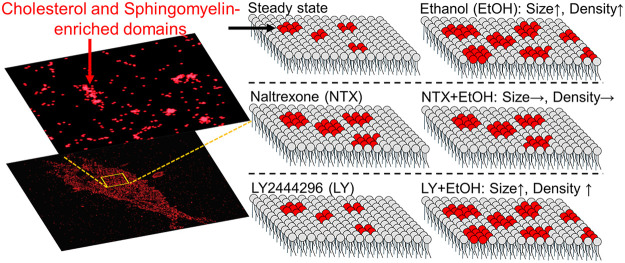

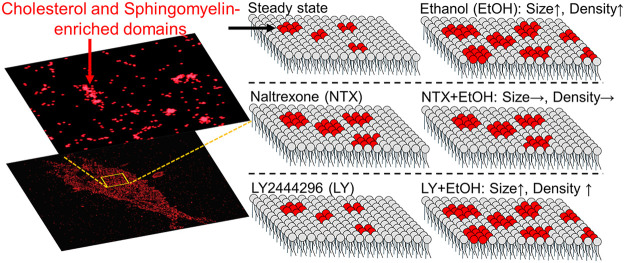

## Introduction

Alcohol (ethanol (EtOH)) is one of the most widely misused substances. Excessive alcohol consumption is harmful and can lead to alcohol use disorder (AUD), a condition with a significant negative impact on the health of the affected individuals, their kith and kin, the society, and the economy.^1,2^ In addition to causing AUD, excessive alcohol use is reported to contribute to over 200 related diseases and deteriorating health conditions^3^ and leads to approximately $249 billion loss to the US economy.^1^ Despite the pressing need for extensive research, our understanding of the detailed molecular mechanisms of EtOH action on cellular physiology remains insufficient. Lack of mechanistic understanding of EtOH effects is in part due to its ubiquitous effects on the function of plasma membrane proteins and lipids, leading to modulation of a variety of neuroendocrine pathways.^4^

Here, our goal is to assess the effects of relevant EtOH concentrations, 10–40 mM, which correspond roughly to a blood alcohol concentration (BAC) of 0.046–0.184%. At the organism level, these BACs relate to states that range from a feeling of being relaxed (≤10 mM) to a state of being heavily inebriated (≥40 mM). We characterized the impact of these EtOH concentrations on the lateral organization and dynamics of cholesterol (Chol)- and sphingomyelin (SM)-enriched domains, a subclass of lipid-enriched plasma membrane domains that often harbor opioid receptors. Opioid receptors, which belong to the superfamily of G protein-coupled receptors (GPCRs), are targets for the general opioid receptor antagonist naltrexone (NTX), one of three pharmacotherapeutics approved for AUD treatment, used to reduce craving for alcohol in abstinent patients and prevent relapse in AUD.^5^ It is also important to emphasize that NTX is not indicated for the management of acute alcohol withdrawal, which often requires benzodiazepines to address neuroexcitability and prevent complications such as seizures. In comparison, the two other U.S. Food and Drug Administration (FDA)- and European Medicines Agency (EMA)-approved medications for treatment of AUD, disulfiram and acamprosate, act through very different biochemical mechanisms. Disulfiram is an aversive agent that produces unpleasant symptoms in patients who consume alcohol by inhibiting the enzyme aldehyde dehydrogenase (ALDH), which is responsible for converting acetaldehyde to acetate during alcohol metabolism.^6^ Acamprosate, i.e., acetyl-homotaurine, is a structural analog of the neurotransmitter gamma-aminobutyric acid (GABA), for which the mechanism of action is still not fully understood. While being administered in high doses, about 2 g a day (e.g., 666 mg three times a day), not metabolized by the liver, and excreted unchanged in urine, it still seems to reach sufficient concentrations in the central nervous system (CNS) to modulate γ-aminobutyric acid type A receptor (GABA_A_) and *N*-methyl-d-aspartate (NMDA) receptor-mediated neurotransmission.^7^

Lipids in the plasma membrane are dynamically organized into distinct lipid phases, the liquid-ordered (L_o_) and liquid-disordered (L_d_) phases.^8^ The liquid-disordered phase is more prevalent and constitutes the majority of the plasma membrane, whereas the liquid-ordered phase comprises small, lipid-enriched domains that are about 10–200 nm in diameter,^9,10^ often referred to as lipid rafts. Lipid rafts are dynamic structures primarily composed of proteins and lipids, Chol and SM in particular,^11^ that are capable of changing their physical properties, such as size, quantity, protein and lipid packing density, in response to internal and/or external stimuli. Furthermore, saturated fatty acids are typically concentrated in the liquid-ordered phase,^12^ where their interactions with Chol lead to the formation of more tightly packed domains and reduced fluidity in comparison with the liquid-disordered phase.^13^ These tightly packed lipid domains help in protein sequestration^14^ and have been viewed as platforms for proteins and their adapters to colocalize and modulate cell signaling.^9,15,16^ Importantly, opioid receptors are often found enriched in these domains.^17^

To study the organization of Chol and SM in ordered domains, we employed two advanced fluorescence techniques: quantitative single-molecule localization microscopy (qSMLM) and fluorescence correlation spectroscopy (FCS), a time-resolved method with single-molecule sensitivity. Due to their single-molecule sensitivity and nanoscale spatial resolution, qSMLM-based techniques are well suited to provide important insights into the lateral organization of plasma membrane-associated molecules at the nanoscale level and their accumulation in/ostracization from lipid rafts.^16,18–20^ To identify lipid rafts, molecular probes such as glycosylphosphatidylinositol (GPI) fused with fluorescent proteins^18,20^ and fluorescently labeled domains of several toxins^21,22^ were used for specific lipid raft targeting and detection. Further, to quantitatively characterize in live cells the concentration and dynamic behavior of molecules within and among lipid rafts, FCS has been used.^17,23^ With a submicrosecond temporal resolution and single-photon detection capacity, FCS records fluctuations in fluorescence intensity that spontaneously arise as fluorescently labeled constituents/molecular probes ramble across the plasma membrane, accruing/dwindling in different regions. The combination of these two approaches provides comprehensive insight into the dynamic lateral organization of the probed lipid molecules in the plasma membrane, allowing us to determine whether, and if so, how it is perturbed by xenobiotic substances such as EtOH and opioid receptor antagonists.

To probe how the lateral organization and dynamics of Chol- and SM- (Chol/SM)-enriched domains are affected by EtOH and selected opioid receptor antagonists, we have used ostreolysin A (OlyA), a ∼15 kDa protein that specifically binds to Chol-complexed SM,^22^ labeled with the Alexa Fluor 647 (AF647) fluorophore (OlyA-AF647). SH-SY5Y cells that naturally express functional opioid receptors: mu-, delta-, and kappa-opioid receptors (MOP, DOP, and KOP, respectively).^24–27^ In particular, we probed how the lateral organization and dynamics of Chol/SM-enriched domains are affected by physiologically relevant concentrations of EtOH (10–40 mM). Additionally, we assessed if treatment with the general opioid receptor antagonist NTX or the kappa-opioid receptor-specific antagonist LY2444296 also perturbs the dynamic lateral organization of Chol/SM-enriched domains and if pretreatment with these opioid receptor antagonists can protect against EtOH-induced effects.

## Methods

### Coverslip Preparation for qSMLM

Coverslips (Fisher Scientific, NC9560650) were cleaned as previously described^28^ and stored until further use in sterile 35 mm tissue culture dishes (Fisher Scientific, 12–565–91) wrapped in aluminum foil. To facilitate cell attachment, the coverslips were coated with the fibronectin-like engineered protein (25 μg/mL, R&D Systems, 1918-FN-02M) in 1× phosphate-buffered solution (PBS) (Gibco PBS (10×), pH 7.4, 70–011–069) immediately before cell seeding.

### Cell Culture

For qSMLM, human neuroblastoma cell line the SH-SY5Y (ATCC, CRL-2266) cells were cultured in phenol red-free DMEM/F12 (Gibco DMEM/F-12, no phenol red, Gibco, 21–041–025) supplemented with 14% fetal bovine serum (FBS; ATCC, 30–2020), 100 U/mL penicillin, and 100 μg/mL streptomycin (Sigma, P0781–100 ML), in a humidified environment at 37 °C and 5% CO_2_. Cells were seeded onto coverslips coated with fibronectin-like engineered protein at a density of around 150,000 cells/coverslip approximately 48 h prior to qSMLM imaging.

For FCS measurements, SH-SY5Y cells were seeded into an 8-well chambered coverglass with a No. 1 borosilicate glass bottom (Thermo Scientific Nunc Lab-Tek, 155411) at a density of 100,000 cells/mL (400 μL/well) approximately 48 h prior to FCS measurements and cultured in DMEM supplemented with 10% FBS, 100 U/mL penicillin, and 100 μg/mL streptomycin.

### Expression and Fluorescence Labeling of OlyA

Plasmids containing C-terminally His-tagged wild-type OlyA (engineered with the following substitutions: C62S, C94S, and S151C) and mutant OlyA (OlyA with the engineered tryptophan to alanine substitution at residue 6 (W6A) that abolishes specific OlyA binding to Chol-complexed SM) in pET-21c­(+) constructs, kindly provided by Dr. A Radhakrishnan,^22^ were transformed into BL21DE3 cells (New England Biolabs). The BL21DE3 cells were grown in LB media with ampicillin (RPI, A40040–100.0) until an optical density of 0.6 was reached, followed by induction with 1 mM isopropyl ß-D-1-thiogalactopyranoside (IPTG) for 16 h at 18 °C. The cells were harvested by centrifugation at 4400 × *g* for 15 min at 4 °C; resuspended in Buffer A (50 mM Tris-HCl (pH 7.5), 150 mM NaCl, 0.4 mg/mL phenylmethylsulfonyl (PMSF) prepared in EtOH, 1 mM tris­(2-carboxyethyl)­phosphine (TCEP), and EDTA-free protease inhibitor cocktail tablet (Sigma, cat. no. S8830)) and lysed with a French press. The lysate was centrifuged at 45,000 × *g* for 1 h at 4 °C using a Ti-70 rotor (Beckman) to pellet the cell debris. The clarified lysate was loaded onto a HisPur Cobalt Superflow Agarose resin (Thermo Scientific, catalog no. 25229) column equilibrated with Buffer B (50 mM Tris-HCl (pH 7.5), 150 mM NaCl, 1 mM TCEP) supplemented with 5 mM imidazole for 30 min at 4 °C on a nutator. The lysate was eluted, and the resin column was washed with 10× the column volume of Buffer B supplemented with 5 mM imidazole. Finally, the C-terminally His-tagged OlyA probe was eluted using Buffer B supplemented with 200 mM imidazole. Protein concentration was determined using the Nanodrop spectrophotometer (Thermo Fisher Scientific), and the protein-rich eluate fractions were pooled and concentrated to about 400 mL. Thereafter, the sample was subjected to gel filtration chromatography (Superdex 6 Increase 10/300 GL in Buffer B; GE Healthcare). Eluate fractions containing the sample with the correct molecular weight were collected and analyzed by SDS-PAGE to confirm purity. Pure C-terminally His-tagged OlyA fractions were aliquoted into cryo vials, flash frozen in liquid nitrogen, and stored at −80 °C for future experiments.

For fluorescence labeling, the purified C-terminally His-tagged OlyA was thawed and spun at 16,000 × *g* for 5 min at 4 °C to pellet any aggregates that may have formed during storage. The sample was transferred to a 1.5 mL Eppendorf tube, and TCEP was added to a final concentration of 1 mM. The sample was incubated with 5 times mole excess of the AF647-maleimide dye (45 min at room temperature on a nutator) followed by 3 times mole excess of the AF647-maleimide dye (45 min at room temperature on a nutator). The reaction was quenched by the addition of 5 times mole excess (against the total dye) of glutathione, followed by a 10 min incubation at room temperature. The reaction mixture was loaded onto hand-packed 0.7 cm × 30 cm chromatography column (Bio-Gel *P*4̅ gel in Buffer C (Buffer B without TCEP); BioRad, cat. no. 1504124) for gravity flow gel filtration chromatography to purify the AF647 fluorescence-labeled C-terminally His-tagged OlyA (OlyA-AF647) from the free AF647 dye. SDS-PAGE was used to confirm the presence of both the OlyA protein (using Coomassie stain) and the conjugated fluorescence AF647 dye (using a LI-COR infrared imager) in the eluate fractions. The total protein concentration and degree of labeling were determined using the NanoDrop spectrophotometer. The fractions that contained OlyA-AF647 were aliquoted, mixed with 20% glycerol, flash frozen, and stored at −80 °C for future experiments.

### OlyA-AF647 Staining of SH-SY5Y Cells

To prepare the OlyA-AF647 probe for cell staining, the stored aliquots were taken out of the −80 °C freezer, allowed to thaw at 4 °C, and centrifuged at 16160 × *g* (i.e., 14 800 rpm) at 4 °C for 5 min to sediment any aggregates that may have formed during storage. Coverslips with SH-SY5Y cells were washed two times with the blocking buffer containing 5% bovine serum albumin (BSA, RPI, A30075–100.0), 0.01% Tween 20 (G-Biosciences, DG011), and 1 mM MgCl_2_ (Fisher Scientific, BP214–500) in 1× PBS. Subsequently, the cells were incubated with 100 mL of 1 μM OlyA-AF647 in blocking buffer for 15 min at room temperature. (Of note, to find an optimal concentration of the OlyA-AF647 probe for staining SH-SY5Y cells, four concentrations were tested: 300 nM, 500 nM, 1 μM, and 3 μM. Cells stained with 1 μM OlyA-AF647 showed a high specific signal with low background, and this concentration was used further in all experiments.) Then, the cells were washed two times with the blocking buffer and four times with 1× PBS. For qSMLM imaging, the cells were fixed by incubating with 4% paraformaldehyde (EMS, 157–8) and 0.2% glutaraldehyde (EMS, 16019) for 30 min at room temperature. The fixative was quenched by incubating the cells with 25 mM glycine (VWR, M103–1KG) for 10 min at room temperature. Finally, the cells were washed three times with 1× PBS. Fluorescent beads (Invitrogen, T7279) were used as fiducial markers for drift correction during analysis. Following fixation, the cells were imaged on the same day. For FCS measurements on live cells, cells stained with OlyA-AF647 were washed out with phenol-red-free DMEM medium and subjected to imaging.

### Photophysical Properties of OlyA-AF647

Photophysical properties of OlyA-AF647 were determined as described before.^29^ Briefly, sparse OlyA-AF647 was attached to cleaned coverslips (less than one molecule in the diffraction-limited spot) and imaged. The average number of appearances of a single fluorescent probe (α) and the maximum dark time were determined.

### Treatment of SH-SY5Y Cells with EtOH, LY2444296, or Naltrexone

To assess the effects of treatment with EtOH (Pharmco, 111000200), naltrexone (NTX; Sigma-Aldrich, N3136–100MG), optical enantiomer of NTX ((+)-NTX; kind gift from Dr. Kenner C. Rice^30^), or LY2444296 (LY; kind gift from Eli-Lilly) on the lateral organization of Chol/SM-enriched domains at the plasma membrane, the following treatment procedures were used.

#### EtOH Treatment

To assess the effects of EtOH concentration, the SH-SY5Y cells were treated with 10 mM, 20 mM, or 40 mM EtOH for 1 h. To assess the effects of the length of exposure to EtOH, the SH-SY5Y cells were treated with 20 mM EtOH for 10 min, 20 min, or 1 h. In all experiments, the SH-SY5Y cells were attached to the coverslips before treatment; EtOH was added to a fresh cell culture medium, and the cells were incubated at 37 °C in a humidified 5% CO_2_ environment. Thereafter, the cells were stained with the adducts of OlyA-AF647 and fixed as outlined above. For FCS measurements on live cells, the cells were stained and washed three times with phenol-red-free FluoroBrite DMEM (Gibco) to remove unbound OlyA-AF647.

#### Naltrexone (NTX) Treatment and Pretreatment

##### Treatment

SH-SY5Y cells were treated with 200 nM NTX in cell culture medium for 1 h at 37 °C in a humidified 5% CO_2_ environment. For qSMLM, the cells were stained with OlyA-AF647 and fixed as outlined above. For FCS measurements on live cells, the cells were stained and washed three times with the phenol-red-free DMEM.

##### Pretreatment

SH-SY5Y cells were treated with 200 nM NTX in cell culture medium for 3 h at 37 °C in a humidified 5% CO_2_ environment. After 3 h incubation, the cells were washed 1 time with 1× PBS (37 °C). Then, the cell culture medium supplemented with 40 mM EtOH was added, and the cells were incubated for 1 h at 37 °C in a humidified 5% CO_2_ environment. After incubation, the cells were stained with OlyA-AF647, fixed as outlined above, and subjected to qSMLM. For FCS measurements, the cells were stained and washed three times with the phenol-red-free DMEM.

The same procedures were applied for (+)-NTX.

#### LY2444296 Treatment and Pretreatment

##### Treatment

SH-SY5Y cells were treated with 100 nM LY2444296 in cell culture medium for 15 min at 37 °C in a humidified 5% CO_2_ environment, then stained with OlyA-AF647, fixed as outlined above, and subjected to qSMLM. For FCS measurements, the cells were stained and washed three times with the phenol-red-free DMEM.

##### Pretreatment

SH-SY5Y cells were treated with 100 nM LY2444296 in cell culture medium for 15 min at 37 °C in a humidified 5% CO_2_ environment. After 15 min of incubation, the cells were washed once with 1× PBS (37 °C). Then, the cell culture medium supplemented with 40 mM EtOH was added, and the cells were incubated for 1 h at 37 °C in a humidified 5% CO_2_ environment. After incubation, the cells were stained with OlyA-AF647, fixed as outlined above, and subjected to qSMLM. For FCS measurements, the cells were stained and washed three times with the phenol-red-free DMEM.

### qSMLM Imaging

qSMLM images were acquired using the N-STORM system (Nikon) with an Eclipse Ti2 inverted microscope and iXON Ultra DU-897U EMCCD camera (other relevant components were described previously).^31^ Laser intensity was ∼85 mW (measured out of the optical fiber from the 640 nm laser). For imaging, 60,000 frames were acquired at an exposure time of 10 ms. For imaging, we used the dSTORM buffer.^32^

### qSMLM Data Analysis

After image acquisition, the raw data files acquired in the nd2 format were processed and converted into text files using NIS Element AR (version 5.21.03). The following settings were used by the software to identify and localize the fluorophore point spread functions into *x*, *y* localization coordinates: 5000 minimum height, 200 nm minimum width, 400 nm maximum width, 300 nm initial fit width, 1.3 max axial ratio, and 1 pixel max displacement. The processed text files were opened using a customized MATLAB (Mathworks, Version R2022a) code, and the drift was corrected using the positions of fiducial beads. Three to four square Regions of Interest (ROIs) with 9–16 μm^2^ size were placed on cell localization data. The average number of localizations per fluorescent probe (α)^29^ was used to determine the number of detected molecules per selected ROI. Pair-correlation (PC) analysis was performed as described before.^20,33,34^ Using values for cluster size (from PC analysis) and a *k*-means-like clustering algorithm,^17,29,33^ we also determined the number of clusters with more than four molecules and the fraction of molecules in those clusters relative to all detected molecules.

### Statistical Analysis of qSMLM Data

Results of the statistical analysis of qSMLM data are summarized in Tables S1–S4. The imaging results for each set of conditions are representative of three independent experiments, with each coverslip containing cells from different passages; no less than 11 cells were imaged per condition. The data were summarized for a given output: detected surface density, reported as the number of detected OlyA-AF647 molecules *per* unit area (molecules/μm^2^); cluster radius (nm); cluster population, reflected by the number of detected OlyA-AF647 molecules *per* cluster (molecules/cluster); and the surface density of clustering, reflected by the number of detected clusters *per* unit area (number of clusters/μm^2^), by reporting the number of ROIs analyzed, median, mean, and standard error of the mean (SEM). To assess sufficient sampling, we randomly split ROIs for each output into two groups and calculated the *p*-value between the two groups (*p*-value_split_)^33^ (Tables S1 and S3). This analysis was carried out using a randomization function followed by splitting and calculation by a two-tailed Student’s *t* test with an unequal variance type in MATLAB. The randomization and *t* test were run 15 times, and the average value was reported. No significant difference was observed between the two groups (*p*-value_split_ ≥ 0.1; Tables S1 and S3). To evaluate the significance of differences between the data of each group output, *p*-values were calculated using Student’s *t* test with two-tailed distribution, two-sample unequal variance (Tables S2 and S4).

### Confocal Laser Scanning Microscopy (CLSM) Imaging and Fluorescence Correlation Spectroscopy (FCS) Measurements in Live SH-SY5Y

CLSM imaging and FCS measurements were performed using the LSM880 (Carl Zeiss, Jena, Germany) microscope system equipped with a 405 nm diode laser, an Ar-laser (458, 488, and 514 nm), 543 and 633 nm HeNe lasers, a water immersion objective (C-Apochromat, 40×, 1.2 N.A., Corr., Carl Zeiss), a gallium arsenide phosphide (GaAsP) detector, and photomultiplier tube (PMT) detectors. AF647 was excited by using the 633 nm laser. The pinhole size was 45 μm (1 Airy unit). The detection wavelength was 650–750 nm. FCS measurements in the plasma membrane were carried out in a series of 10 consecutive measurements, with each measurement lasting 20 s.

### FCS Data Analysis

FCS data were analyzed using ZEN software (Carl Zeiss, Jena, Germany). The autocorrelation function, *G*(τ), was calculated as

$$G(\tau )=\frac{\left\langle I(t)\times I(t+\tau )\right\rangle }{\langle I(t)\rangle^{2}}$$ 1

where τ denotes the lag time, *I*(*t*) is the fluorescence intensity at time *t*, and chevron brackets denote average values of the analyzed variable over time. The autocorrelation curves (ACCs) are plots of *G*(τ) as a function of lag time τ.

The ACCs were fitted using an analytical function for concomitant three-dimensional (3D) and two-dimensional (2D) diffusion of one species, the so-called two-component model with free 3D diffusion of the component designated as component 1, and free 2D diffusion of the component designated as component 2:

$$G(\tau )=1+\left(1+\frac{F_{T}\times e^{-\tau /\tau_{T}}}{1-F_{T}}\right)\times \frac{1}{N}\times \left[F_{1}\times {\left(1+\frac{\tau }{\tau_{D1}}\right)}^{-1}\times {\left(1+\frac{\tau }{S^{2}\times \tau_{D1}}\right)}^{-1/2}+(1-F_{1})\times {\left(1+\frac{\tau }{\tau_{D2}}\right)}^{-1}\right]$$ 2a

In eq 2a, *F*_T_ is the average fraction of the AF647 fluorophore in the triplet state; τ_T_ is the average relaxation time of the triple state; τ_D1_ and τ_D2_ are the average diffusion times of the first and second component, respectively; *N* is the average total number of OlyA-AF647 molecules in the effective volume element; and *F*_1_ is the mole fraction of the first component, i.e. the unbound OlyA-AF647 fraction that remained in the cell culture medium without binding to the plasma membrane. *S* is the so-called structure parameter of the effective volume element, $S=\frac{\omega_{z}}{\omega_{xy}}$ . To quantify using eq (2*a*) the OlyA-AF647 diffusion time in the plasma membrane (τ_D2_), the diffusion time of OlyA-AF647 in the medium (τ_D1_) was fixed to the value measured in the cell culture medium alone (τ_D1_ = 213 μs).

Values of the lateral (ω_*xy*_) and axial (ω_*z*_) radii of the effective volume element, i.e., the structure parameter *S*, were determined by calibration using as a reference the AF647 dye for which the diffusion coefficient is known (*D*_AF647_; 330 μm^2^s^–1^).^35^ To this aim, the ACC acquired in calibration experiments using a diluted solution of the AF647 dye of known concentration was fitted using an analytical function for free 3D diffusion of a single species (so-called one-component model):

$$G(\tau )=1+\left(1+\frac{F_{T}\times e^{-\tau /\tau_{T}}}{1-F_{T}}\right)\times \frac{1}{N}\times {\left(1+\frac{\tau }{\tau_{\mathrm{DAF}647}}\right)}^{-1}\times {\left(1+\frac{\tau }{S^{2}\times \tau_{\mathrm{DAF}647}}\right)}^{-1/2}$$ 2b

to determine *F*_T_, τ_T_, *N*, τ_DAF647_, and *S*. From these values, the lateral and axial radii were determined:

$$\omega_{xy}=(4\times D_{\mathrm{AF}647}\times \tau_{\mathrm{AF}647}{)}^{1/2}$$ 3

$$\omega_{z}=\omega_{xy}\times S$$ 4

The diffusion coefficient of OlyA-AF647 in the plasma membrane was calculated using the diffusion time of OlyA-AF647 on the plasma membrane (τ_D2_ in eq 2a) and the lateral radius (ω_*xy*_) was determined using eq 3.

Apparent density of OlyA-AF647 in the plasma membrane (ρ_OlyA–AF647_^membrane^) and apparent brightness of OlyA-AF647, which is reflected by counts *per* second *per* molecule (CPM), were assessed as follows:

$$\rho_{\mathrm{membrane}}^{\mathrm{OlyA}‐\mathrm{AF}647}=\frac{F_{2}\times N}{\omega_{xy}^{2}}$$ 5

$$\mathrm{CPM}=\frac{I}{N}$$ 6

where *F*_2_ (*F*_2_ = 1 – *F*_1_) is the membrane-bound fraction of OlyA-AF647 and *N* is the number of molecules determined by fitting eq 2a to the experimentally derived autocorrelation curves, and *I* is the average fluorescence intensity measured in a 20 s time series.

To evaluate the difference between diffusion coefficients or densities measured under different treatments as compared to the corresponding values measured in untreated cells, statistical analysis was performed using the nonparametric Mann–Whitney test routine available in the Origin Data Analysis and Graphing Software 2019 (OriginLab Corporation, USA).

### Calcium Imaging

SH-SY5Y cells were seeded into 8-well chambered coverglass as described above. The cells were stained with 2 μM Fura Red in phenol-red-free FluoroBrite DMEM with 0.1% Pluronic F-127 for 30 min. After washing the cells twice with the phenol-red-free FluoroBrite DMEM medium, the cells were treated as follows: with 200 nM NTX for 1 h; or with 100 nM LY2444296 for 15 min; or with 10 mM/40 mM EtOH for 1 h; or with 200 nM NTX for 3 h/100 nM LY for 15 min followed by EtOH for 1 h as described above. After the treatment, the cell culture medium was replaced with 200 μL of Dulbecco’s phosphate-buffered saline (DPBS(−); Gibco: 14190–144) with 1.8 mM CaCl_2_. During the time-lapse imaging, the first 4 images were acquired before the K^+^ depolarization, followed by 10 min acquisitions after depolarization using 200 μL of DPBS(−) with 5.0 mM CaCl_2_ and 90 mM KCl.

For calcium imaging, the same microscope system, LSM880 (Carl Zeiss, Jena, Germany), was used as for CLSM and FCS, but with a different optical setting. Here, the 405 nm diode laser and the Ar-laser (458, 488, and 514 nm); the water immersion objective (C-Apochromat, 40×, 1.2 N.A., Corr, Carl Zeiss); and the gallium arsenide phosphide (GaAsP) detector and the photomultiplier tube (PMT) detectors were used. Free, i.e., Ca^2+^-unbound Fura Red was excited using the 488 nm laser line of the Ar-laser. The Ca^2+^-bound Fura Red was excited using the 405 nm laser. To minimize crosstalk, the multitrack mode was used. The optical setting for Ca^2+^-unbound Fura Red imaging was: 488 nm excitation, 650–740 nm detection, 600 μm pinhole. The optical setting for Ca^2+^-bound Fura Red was: 405 nm excitation, 650–740 nm detection, 90 μm pinhole. 55 CLSM images were collected during 10 min, of which the first 4 images were acquired before and 51 images after K^+^ depolarization using the depolarization buffer containing 5.0 mM CaCl_2_ and 100 mM KCl in DPBS(−).

To account for the photobleaching of Fura Red during time-lapse fluorescence imaging, we calculated the Fura Red ratio (FRr).

$$\mathrm{FRr}(t)=\frac{I(t{)}_{\mathrm{Ca}‐\mathrm{bound}}}{I(t{)}_{\mathrm{Ca}‐\mathrm{unbound}}}$$ 7

where *I*(*t*)_Ca‑bound_ and *I*(*t*)_Ca‑unbound_ are the fluorescence intensities of Ca^2+^-bound and Ca^2+^-unbound Fura Red, respectively. The amplitude and the Fura Red ratio (FRr) at the baseline were analyzed in each cell.

To evaluate the difference between the baseline or amplitude of FRr measured under different treatments as compared to the corresponding values measured in untreated cells, statistical analysis was performed using the nonparametric Mann–Whitney test routine available in the Origin Data Analysis and Graphing Software 2019 (OriginLab Corporation, USA).

### Plasma Membrane Polarizability/Fluidity Measurement

The fluorigenic and solvatochromic plasma membrane-targeting dye MemGlow NR12S (Cytoskeleton Inc., Denver, Colorado, USA) that exhibits a wavelength shift upon binding to plasma membranes in different phases, Lo or Ld, was used to characterize plasma membrane polarizability/fluidity. To this aim, 4 nmol of NR12S was dissolved in dimethyl sulfoxide (DMSO) to prepare a 20 μM stock solution. Stock solution aliquots were stored at −20 °C. Before use, a 200 nM NR12S working solution was prepared by diluting the NR12S stock solution with phenol-red-free FluoroBriteTM DMEM (Gibco). The cells were treated as follows: with 200 nM NTX for 1 h; 200 nM/100 μM (+)-NTX for 1 h; or with 100 nM LY2444296 for 15 min; or with 40 mM EtOH for 1 h; or with 200 nM NTX for 3 h/200 nM or 100 μM (+)-NTX for 3 h/100 nM LY for 15 min followed by 40 mM EtOH for 1 h as described above. Following treatment, SH-SY5Y cells were stained for 10 min by using the NR12S working solution. Plasma membrane polarizability was measured using the same LSM880 microscope system as for CLSM and FCS, and the following optical settings. NR12S was excited by using the 543 nm laser. Fluorescence was detected in the multitrack mode, using the same GaAsP detector to record fluorescence intensities at 560–580 and 640–660 nm. The pinhole size was 40 μm (1 Airy unit).

The ZEN software was used to make line profiles across the plasma membrane of NR12S. Maximum fluorescence intensity in the line profile yielded the fluorescence intensity at the plasma membrane. General polarizability (GP) was calculated as follows:^36^

$$\mathrm{GP}=\frac{I_{560-580}-I_{640-660}}{I_{560-580}+I_{640-660}}$$ 8

where *I*_560–580_ and *I*_640–660_ are the fluorescence intensities recorded at wavelengths 560–580 nm and 640–660 nm, respectively, which are fluorescence emission maxima of NR12S in the Lo and the Ld phases, respectively. For each cell, the GP value was determined as an average of 4–10 different positions at the plasma membrane. Smaller GP values indicate higher membrane fluidity, i.e., lower Chol levels, and *vice versa*.

## Results

### OlyA-AF647 Binds Specifically to Chol-Complexed SM in SH-SY5Y Cells

Wild-type OlyA (OlyA) and mutant OlyA (MT-OlyA)^22^ were purified using affinity chromatography followed by size exclusion chromatography (Figure S1A)^37^ and subsequently labeled with the maleimide functionalized fluorescent dye Alexa Fluor 647 (AF647) through conjugation to the single cysteine, Cys151. The free dye was removed by using gravity flow size exclusion chromatography. Eluted aliquots were run on SDS-PAGE to confirm the presence and purity of protein (using Coomassie stain) and fluorescence dye conjugation (using a LI-COR infrared imager) (Figure S1B).^37^ The degree of labeling of both OlyA-AF647 and MT-OlyA-AF647 was typically between 0.7 and 0.8, depending on the aliquot. Using previously established methodology,^29^ we have characterized the photophysical properties of this fluorescent probe. The average number of localizations of a single OlyA-AF647 fluorescent probe was α = 3.3, and the maximum dark time was 250 s (Figure S2). We then examined different conditions for SH-SY5Y cell staining with OlyA-AF647 (Figure S3). We identified 15 min incubation using 1 μM OlyA-AF647 as the staining condition with the saturated detected density on the cell surface without the effect on background noise. Finally, we confirmed that under these staining conditions, MT-OlyA-AF647 does not bind to the SH-SY5Y cells (Figure S4).

### EtOH Alters the Nanoscale Organization of Chol/SM-enriched Domains at the Plasma Membrane in a Concentration- and Treatment Time-Dependent Manner

SMLM images of untreated and EtOH-treated SH-SY5Y cells show dense clusters of Chol-complexed SM together with abundant smaller nanodomains at the basal plasma membrane (Figure 1A). Compared to steady state, quantitative data analysis indicates that 1 h treatment with 10 mM EtOH: (1) did not significantly affect the detected surface density (Figure 1B); (2) significantly increased the cluster size (Figure 1C); (3) did not significantly alter the cluster population (Figure 1D); (4) did not significantly alter surface density of clusters (number of clusters *per* μm^2^; Figure 1E). However, compared to steady state, 1 h treatment with 20 or 40 mM EtOH led to significantly increased values of all four characteristics; there was no statistically significant difference in any of them for 1 h treatments using 20 *vs* 40 mM EtOH (Figure 1B–E, Tables S1 and S2).

**Figure 1:**
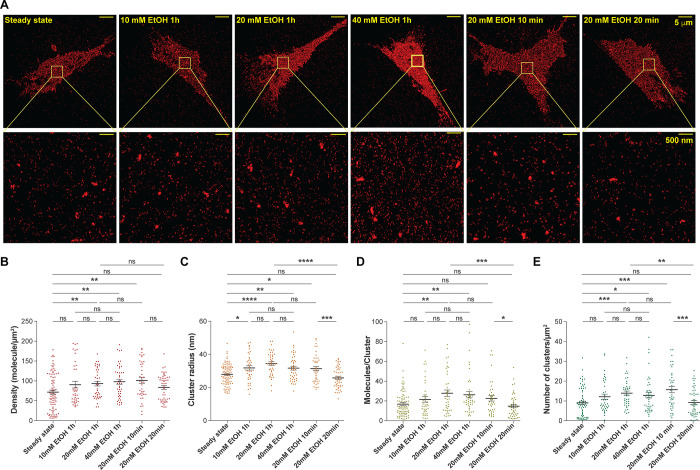
EtOH alters the nanoscale organization of Chol/SM-enriched domains at the plasma membrane of SH-SY5Y cells. (A) qSMLM images of representative cells (upper row) with details shown in magnified regions (lower row). (B) Detected surface density in untreated and EtOH-treated cells. (C) Cluster radii. (D) Detected molecules *per* cluster. (E) Number of detected clusters *per* μm^2^. For panels (B–E), dots represent respective values for investigated regions of interest (ROIs), lines represent the mean value of the measured variable, and short error bars represent standard error of the mean (SEM). The number of replicates *n* = 3 signifies independent experiments *per* condition; numerical values for all measured variables and statistical significance *p*-values are provided in Tables S1 and S2.

Interestingly, we have observed time-dependent effects for treatment with 20 mM EtOH. Under these conditions, the detected surface density (Figure 1B), cluster radius (Figure 1C), and the surface density of clustering (Figure 1E), transiently increased after 10 min of EtOH treatment, returned to steady state values after 20 min of EtOH treatment, and then increased again, all reaching significantly higher values compared to steady state for 1 h of EtOH treatment (Figure 1B–E, Tables S1 and S2). Compared to the steady state, the cluster population (number of molecules in clusters) did not significantly change at 10 and 20 min but significantly increased after 1 h treatment 20 mM EtOH (Figure 1D, Tables S1 and S2).

### Pretreatment with NTX Guards against EtOH-Induced Nanoscale Reorganization of Chol/SM-Enriched Domains At the Plasma Membrane

qSMLM imaging revealed that treatment with both 200 nM NTX and 100 nM LY2444296 affected the lateral organization of Chol/SM-enriched domains. NTX treatment led to an increase in detected surface density and clustering surface density, whereas LY2444296 treatment led to only a decrease in cluster population (Tables S3 and S4).

Moreover, qSMLM imaging (Figure 2) showed that pretreatment with 200 nM NTX fully prevents the EtOH-induced reorganization of Chol-complexed SM, as evident from the lack of change in the detected surface density (Figure 2B), cluster radius (Figure 2C), number of molecules per *per* cluster (Figure 2D), and density of Chol/SM clusters (Figure 2E). In contrast, pretreatment with 100 nM LY2444296 had no preventive effects, as reflected by a significant increase in all characterized properties of Chol/SM**-**enriched domains (Figure 2B–E, Tables S3 and S4).

**Figure 2:**
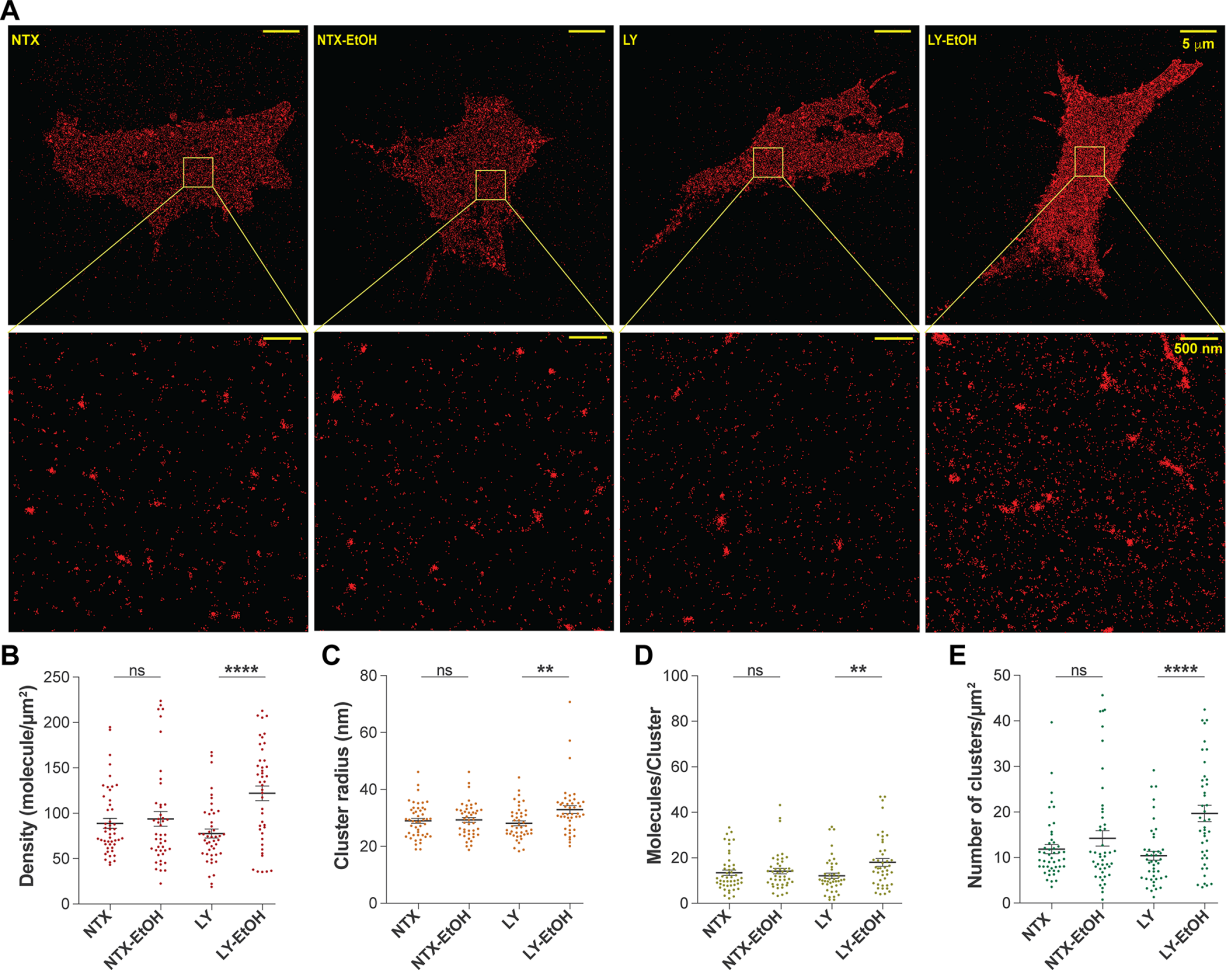
Pretreatment with NTX guards against EtOH-induced nanoscale reorganization of Chol/SM-enriched domains at the plasma membrane. (A) qSMLM images of representative cells (upper row) with details shown in magnified regions (lower row). (B) Detected surface densities. (C) Cluster radii. (D) Detected molecules *per* cluster. (E) Number of detected clusters per μm^2^. For panels (B–E), dots represent respective values for investigated regions of interest (ROIs), long lines represent the mean value of the measured variable, and short error bars represent standard error of the mean (SEM). The number of replicates *n* = 3 signifies independent experiments *per* condition; numerical values for all measured variables and statistical significance *p*-values are provided in Tables S3 and S4.

### Pretreatment with NTX Guards against EtOH-Induced Changes in the Lateral Mobility of Chol-Complexed SM in the Plasma Membrane

CLSM imaging clearly showed the plasma membrane localization of OlyA-AF647 in live SH-SY5Y cells (Figure 3A). Using diffraction-limited imaging, differences in OlyA-AF647 localization were not observed under any of the tested treatments (Figure S5A). Time-resolved fluorescence intensity fluctuations recorded at the apical plasma membrane of untreated SH-SY5Y cells showed markedly larger amplitudes compared to the amplitude of fluctuations recorded in the cell culture medium, as is evident from the fluorescence intensity time series normalized to their own average count rate (Figure 3B, upper vs lower time series). The significantly longer decay time of the ACCs recorded in the plasma membrane as compared to that of ACCs recorded in the cell culture medium (Figure 3C, black vs gray ACCs) clearly indicates binding of OlyA-AF647 to Chol-complexed SM and its significantly slower lateral diffusion at the plasma membrane as compared to that in the cell culturing medium.

**Figure 3:**
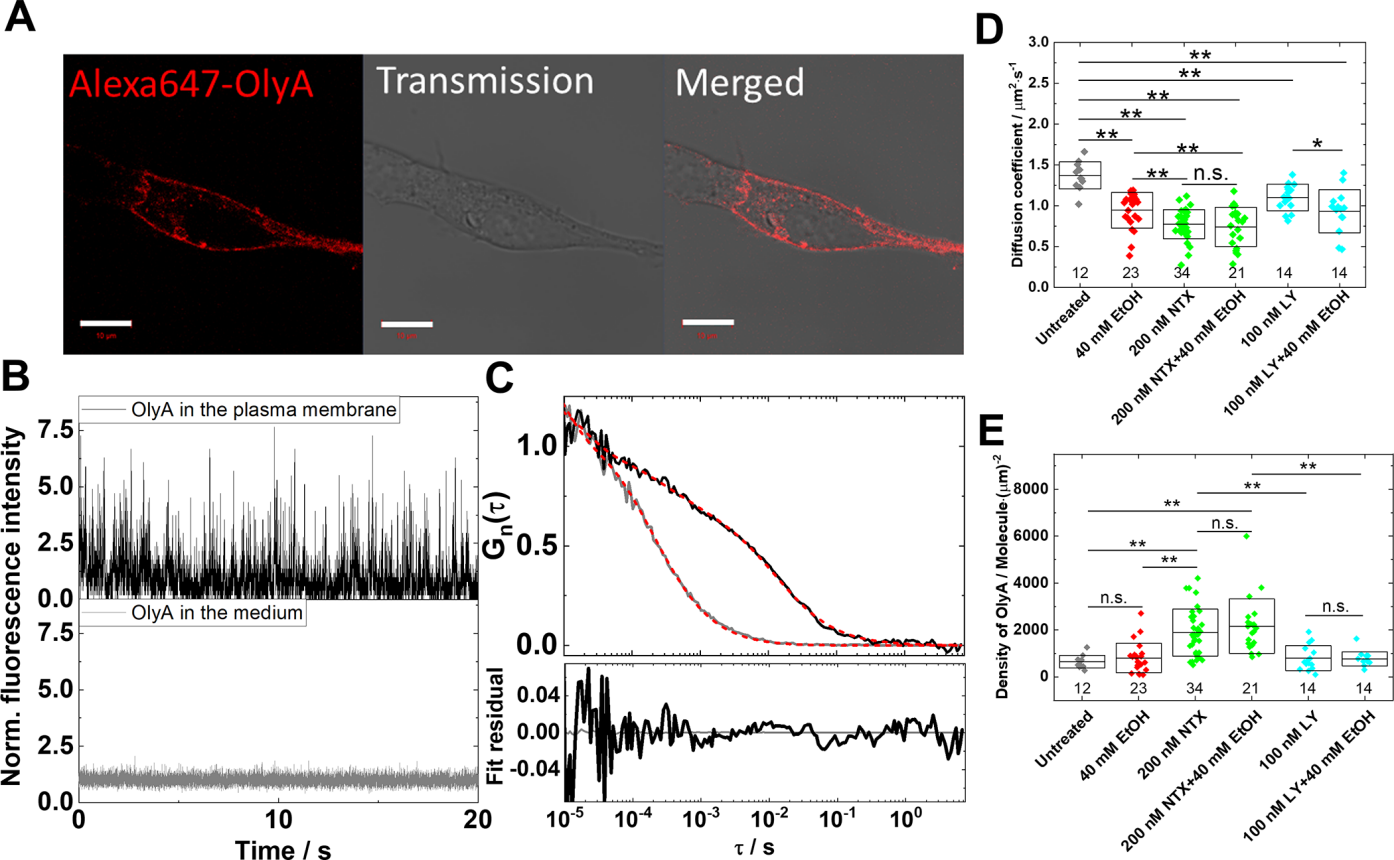
Pretreatment with NTX guards against EtOH-induced changes in the lateral mobility of Chol/SM-enriched domains and the surface density of Chol/SM-complexes in the plasma membrane of live cells. (A) CLSM image of a live SH-SY5Y cell fluorescently labeled with OlyA-AF647 shows plasma membrane localization of the Chol/SM-complexes-specific OlyA-AF647 probe. (B) Fluorescence intensity fluctuations normalized by corresponding average fluorescence intensities, CR_Medium_ = 52 kHz and CR_pl.memb._ = 3.5 kHz. (C) Autocorrelation curves (ACCs; top) and fit residuals (bottom) revealing stark differences between OlyA-AF647 movement in the cell culture medium (gray) and in the plasma membrane (black). (D, E) Diffusion coefficient of the OlyA-AF647 probe specifically bound to Chol/SM-complexes (D) and surface area density (E), in untreated cells and under the treatment with 40 mM EtOH, 200 nM NTX, 200 nM NTX + 40 mM EtOH, 100 nM LY2444296 (LY) or 100 nM LY2444296 + 40 mM EtOH. The values are given as average ± standard deviation. The numbers shown below the data points indicate the number of analyzed cells. Statistical analysis was performed using the nonparametric Mann–Whitney test. Asterisks indicate a statistically significant difference, with *p*-values *p* < 0.05 (*) and *p* < 0.01 (**); *n.s.* denotes not statistically significant.

Treatment with 40 mM EtOH, 200 nM NTX, or 100 nM LY2444296 significantly lowered the mobility of Chol-complexed SM in the plasma membrane, as reflected by the statistically significant decrease in the diffusion coefficient of the OlyA-AF647 probe (Figure 3D,Figure S5B–D). In addition, a statistically significant increase in the surface area density of OlyA-AF647 in the OVE was observed following treatments with 200 nM NTX but not following treatment with 40 mM EtOH or 100 nM LY2444296 (Figure 3E). The average brightness of the OlyA-AF647 probe, as reflected by the CPM, is not altered by the treatments themselves (Figure S6).

Importantly, pretreatment of the cells with NTX was protective against EtOH-induced effects on OlyA-AF647 diffusion, as reflected by the lack of statistically significant difference (Figure 3D, green), whereas pretreatment with LY2444296, at the concentration and treatment time tested, was not (Figure 3D, cyan).

### Pretreatment with NTX Guards against EtOH-Induced Changes in Plasma Membrane Fluidity

Treatment with 40 mM EtOH significantly increased plasma membrane fluidity, characterized using fluorigenic and solvatochromic plasma membrane-targeting dye NR12S (Figure 4A), as reflected by the statistically significant decrease in GP (Figure 4B). While neither NTX, (+)-NTX, nor LY2444296 altered plasma membrane fluidity on their own (Figure 4B, green, cyan, or navy blue vs black), pretreatment with 200 nM NTX warded of EtOH-induced changes in plasma membrane fluidity (Figure 4B, green vs blue), while pretreatment with 100 nM LY2444296 did not show this effect (Figure 4B, navy blue vs violet). Pretreatment with 200 nM (+)-NTX, the optical enantiomer of NTX that is nearly devoid of opioid receptor binding and dissociation constants in the μM range, was not protective against EtOH-induced changes in plasma membrane fluidity (Figure 4B, cyan vs magenta), suggesting that opioid receptor-mediated mechanisms are important for the observed protective effects of NTX against EtOH-induced changes in plasma membrane fluidity. In line with this reasoning, 100 μM (+)-NTX protected against EtOH-induced reduction in plasma membrane GP, i.e., an increase in plasma membrane fluidity (Figure 4B, red vs dark yellow).

**Figure 4:**
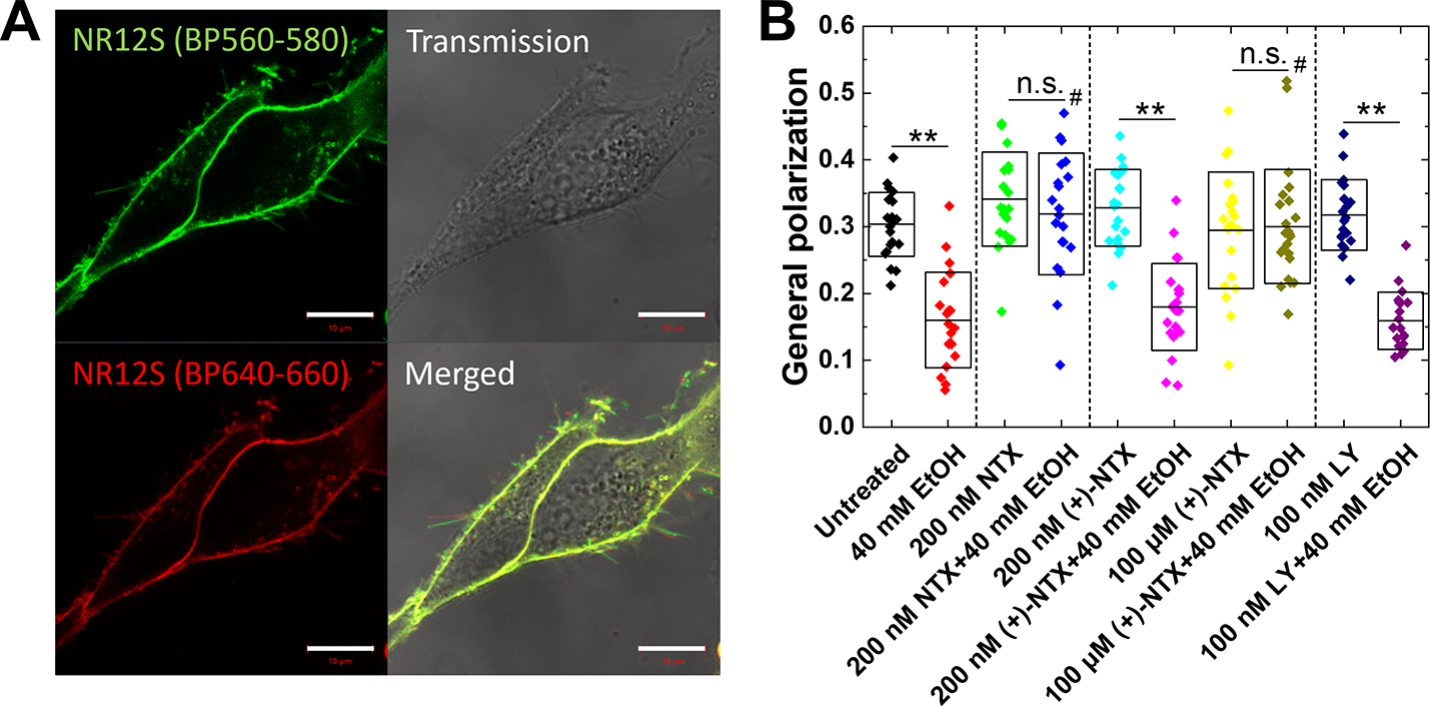
Pretreatment with NTX guards against EtOH-induced alterations in plasma membrane fluidity in SH-SY5Y cells. (A) Confocal laser scanning microscopy images of live SH-SY5Y cells stained with NR12S acquired using two different band-pass filters: BP560–580 (green) and BP640–660 (red). Scale bar: 10 μm. (B) General polarization (GP) values for individual NR12S-stained cells (dots), and whiskers plot showing confidence intervals, average ± standard deviation, in each group. Lower GP values reflect higher plasma membrane fluidity. Black: untreated, red: 40 mM EtOH, green: 200 nM NTX, blue: 200 nM NTX + 40 mM EtOH, cyan: 200 nM (+)-NTX, magenta: 200 nM (+)-NTX + 40 mM EtOH, yellow: 100 μM (+)-NTX, dark yellow: 100 μM (+)-NTX + 40 mM EtOH, navy blue: 100 nM LY2444296, violet: 100 nM LY2444296 + 40 mM EtOH. Statistical analysis was performed using one-way ANOVA test with *Tukey* test. Asterisks indicate statistically significant difference with respect to untreated cells, *p* < 0.05 (*), *p* < 0.01 (**). Hashtag indicates statistically significant difference with respect to cells treated with 40 mM EtOH, *p* < 0.01 (#). *n.s.* denotes not statistically significant.

### Pretreatment with NTX Suppresses EtOH-Induced Ca^2+^ Elevation in Response to K^+^-Induced Depolarization in Live SH-SY5Y Cells

Time-lapse ratiometric calcium ion (Ca^2+^) imaging using the red fluorescent Ca^2+^ indicator Fura Red (Figure 5A), shows a K^+^-induced increase of Ca^2+^-bound Fura Red (Figure 5B, green) and decrease of Ca^2+^-unbound Fura Red (Figure 5B, red). To account for the effects of cell movement/shrinkage and/or Fura Red photobleaching during CLSM imaging, the Fura Red ratio (FRr), i.e., the ratio of Ca^2+^-bound vs Ca^2+^-unbound Fura Red fluorescence, was calculated for each cell and its change was monitored over time (Figure 5C). Our data show that FRr transiently increases upon stimulation with K^+^ (Figure 5C), reflecting an abrupt increase in Ca^2+^ concentration after K^+^-induced depolarization of SH-SY5Y cells, and subsequently gradually decays as the cell repolarizes and the intracellular Ca^2+^ level returns to the baseline value. To quantify the transient increase in Ca^2+^ concentration, we have assessed the FRr at the baseline, before depolarization induction (Figure 5D), which is proportional to baseline intercellular Ca^2+^ levels; and the amplitude of the FRr, which reflects the extent of change in intercellular Ca^2+^ levels under depolarization (Figure 5E).

**Figure 5:**
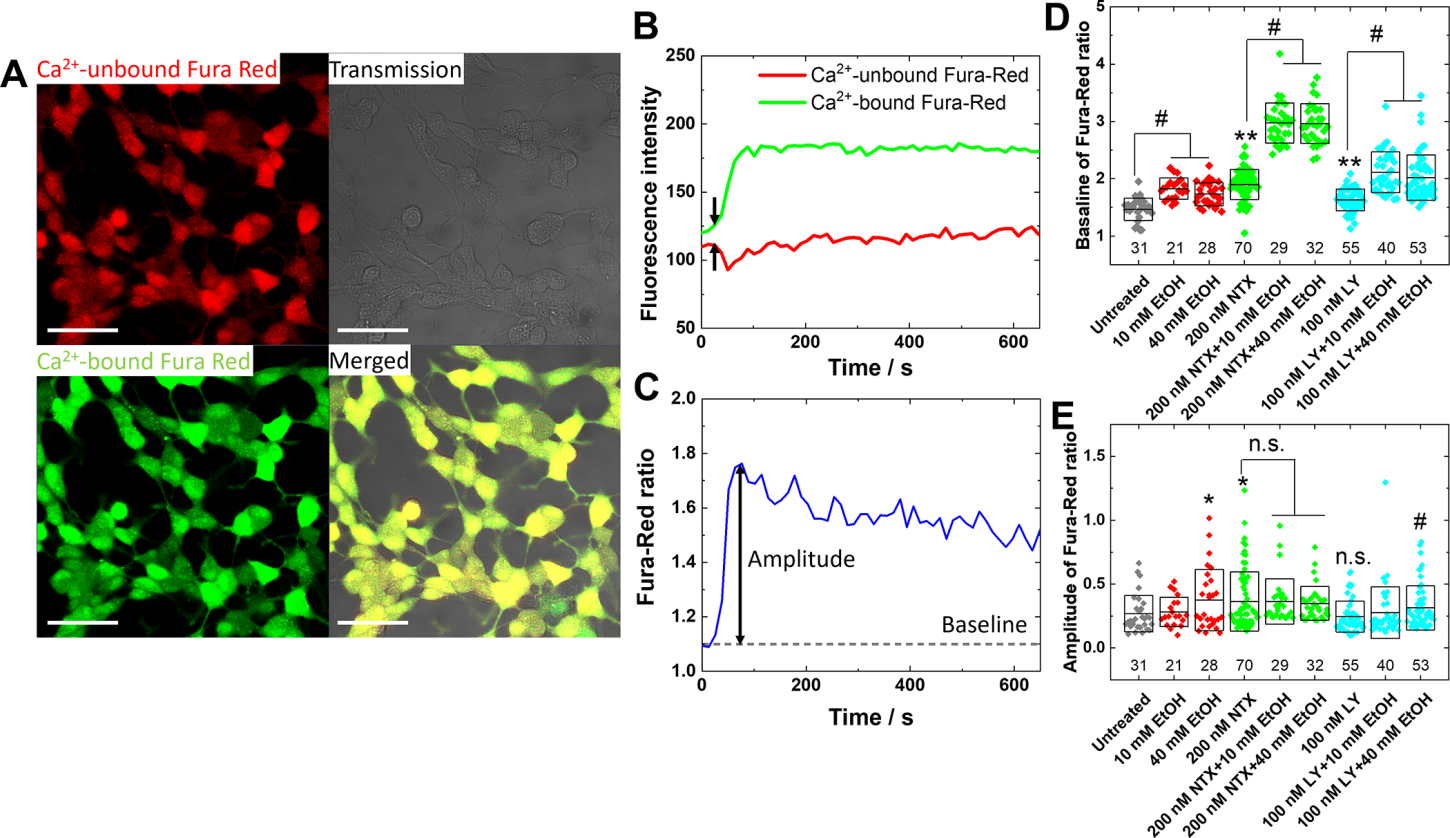
Effect of EtOH and treatment/pretreatment with selected opioid receptor antagonists on EtOH-induced intracellular Ca^2+^ elevation in response to K^+^-induced depolarization in live SH-SY5Y cells. (A) CLSM images showing Ca^2+^-unbound Fura Red (red) and Ca^2+^-bound Fura Red (green) distribution in live, untreated SH-SY5Y cells (gray, transmission image) and all channels superimposed on one another (merged). Scale bar: 50 μm. (B) Changes in Ca^2+^-unbound Fura Red (red) and Ca^2+^-bound Fura Red (green) fluorescence monitored in untreated SH-SY5Y cells by time-lapse imaging. Arrow indicates the instant of K^+^-induced depolarization. (C) FRr calculated from B by dividing the Ca^2+^-bound Fura Red fluorescence intensity at time *t* with Ca^2+^-unbound Fura Red fluorescence at the same point in time. The amplitude of FRr, defined as the difference in FRr at the first maximum after K^+^-induced depolarization and the average FRr at baseline, i.e., before K^+^-induced depolarization, and FRr at baseline were analyzed in each cell. (D) Average ± standard deviation of FRr at baseline. The number of single cells analyzed is indicated below. Statistical analysis was performed using the nonparametric Mann–Whitney test. Asterisks indicate statistically significant difference with respect to untreated cells, with *p*-values *p* < 0.01 (**). Hashtag indicates statistically significant difference with respect to cells treated with the corresponding antagonist, *p* < 0.01 (#). *n.s.* denotes not statistically significant. E. Average ± standard deviation of the amplitude of FRr normalized by the FRr at baseline. The number of single cells analyzed is indicated below. Statistical analysis was performed using the nonparametric Mann–Whitney test. Asterisk indicates a statistically significant difference with respect to untreated cells, *p* < 0.05 (*). Hashtag indicates statistically significant difference with respect to cells treated with 100 nM LY2444296, *p* < 0.01(#). *n.s.* denotes not statistically significantly different in comparison between the effects of treatment with 200 nM NTX and 200 nM NTX with 10 mM/40 mM EtOH, or between the effects of treatment with 100 nM LY2444296 compared to untreated cells.

Furthermore, the FRr at baseline is significantly higher in cells pretreated with EtOH, indicating that intercellular Ca^2+^ levels are elevated under EtOH treatment (Figure 5D, red vs gray). Unexpectedly, significantly increased FRr at baseline was also observed in cells treated with NTX or LY2444296 (Figure 5D, green vs gray and cyan vs gray, respectively), indicating that intercellular Ca^2+^ levels were elevated in these cells as well. The response to K^+^-induced depolarization showed that the amplitude of the increase in FRr was higher in cells treated with higher doses of EtOH, showing no significant differences for cells treated with 10 mM EtOH and a statistically significant increase in amplitude in cells treated with 40 mM EtOH (Figure 5E, red vs gray). Pretreatment with NTX but not with LY2444296 mitigated the effect of 40 mM EtOH, yielding an insignificant difference in the amplitude of FRr (Figure 5E, green vs gray) while the difference in the amplitude of FRr in cells pretreated with LY2444296 was statistically significantly different for treatment with 40 mM EtOH (Figure 5E, cyan vs gray).

## Discussion

Endogenous opioid receptors dynamically organize into nanodomains at the plasma membrane of cells and at least partially associate with Chol/SM-enriched domains, i.e., lipid rafts.^17^ These nanoscale organizational features likely affect their coupling to G-proteins, thus affecting downstream signaling.^38^ To develop new pharmacotherapeutics for AUD, it is therefore essential to understand how EtOH and approved and prospective candidates for AUD treatment affect the dynamic lateral organization and sorting of plasma membrane lipids and plasma membrane receptors into Chol/SM-enriched domains.

We have previously shown that EtOH at concentrations that have physiological effects in humans, 10–40 mM, significantly affects the lateral nanoscale organization of plasma membrane constituents:^18,39^ increasing the density of the lipid raft marker glycosylphosphatidylinositol (GPI);^18^ increasing the size and the occupancy of GPI nanodomains;^18^ increasing KOP but not MOP surface density;^39^ and decreasing the size and occupancy of opioid receptor-harboring nanodomains.^39^ Here we show that EtOH also profoundly affects the lateral organization and dynamics of Chol/SM-enriched domains.

Most notably, 1 h treatment with the lowest EtOH concentration tested (10 mM) increased only the size of Chol/SM-enriched domains, while 1 h treatments with 20 or 40 mM EtOH increased the detected surface density and properties of Chol/SM-enriched domains (size, molecules per cluster, and clustering density; Figure 1B, Tables S1 and S2). Interestingly, we have observed that treatment with 20 mM EtOH showed a transient increase of detected surface density, cluster radius, and clustering density, with increasing values observed 10 min after EtOH addition, returning to steady state values at 20 min, and then increasing again after 1 h (Figure 1B–E, Tables S1 and S2). The transient rearrangement of lateral lipid organization observed by qSMLM may explain the finding that acute EtOH challenge nearly instantaneously, within seconds after exposure, enhances the activity of the large conductance Ca^2+^ and voltage-gated K^+^ (BK_Ca_) channel, causing an effect that thereafter subsides within minutes.^40^ Importantly, our results are also in line with findings that the sensitivity of the BK_Ca_ channel to EtOH challenge can be mediated by Chol,^41^ and that short- and long-term exposure to EtOH exert different effects on plasma membrane lipid composition and cell surface receptor functions, as reviewed by Pietrzykowski and Treistman.^42^ Additionally, the EtOH-induced increase in cluster radius and cluster population that are observed with qSMLM upon 1 h treatment with 40 mM EtOH (Figure 1C, Tables S1 and S2) is in agreement with the significant lowering in diffusion coefficients and the increase in average total number of OlyA-AF647 molecules in the effective volume element that is observed by FCS (Figure 3D, Table S5). The observed agreement between qSMLM and FCS measurements demonstrates the robustness of our measurements and strengthens the validity of our conclusions.

Moreover, our data show that treatment with both NTX and LY2444296 significantly slowed down the diffusion of OlyA-AF647 bound to Chol/SM-enriched domains (Figure 3D,Figure S5B–D). Given that treatment with these antagonists alone did not change the size of these domains (Tables S3 and S4) nor bulk plasma membrane fluidity (Figure 4B), this may indicate that antagonist-induced changes in local biophysical properties of the plasma membrane, such as increased nanoscale plasma membrane viscosity at Chol/SM-enriched domains, may have occurred. The capacity of the investigated opioid antagonists to affect compartmentalization in the plasma membrane via both cell surface receptor-cytoskeleton-based and/or lipid-dependent mechanisms, which are the two main compartmentalizing forces at work in the plasma membrane, provides important new insights into their mechanism of action.

We have previously shown that the general opioid receptor antagonist NTX exerted distinct effects on MOP and KOP nanodomains organization and protected against ethanol-induced MOP and KOP reorganization.^39^ In the present study, we show that NTX also changed Chol/SM detected density, cluster populations, and cluster density. Importantly, NTX also showed a profound protective effect against EtOH-induced rearrangement of Chol/SM-enriched domains, consistent with our previous findings that pretreatment with NTX guards against EtOH-induced rearrangement of GPI, MOP, and KOP lateral organization in the plasma membrane.^18,39^ In contrast, the KOP-specific antagonist LY2444296, at the concentration and treatment time tested, did not show such a protective effect, although it did induce changes in the Chol/SM cluster populations. This is not unexpected for several reasons. First, LY2444296 is KOP-selective, whereas NTX is a general opioid receptor antagonist that binds all opioid receptors, with the highest affinity for MOP. Given that SH-SY5Y cells endogenously expresses more MOP than KOP^26,27,43^ and that functional studies report only a ∼10% inhibition of forskolin-stimulated adenylate cyclase activity by the KOP-selective agonist U-50488H, compared to the effect of MOP- and DOP-selective ligands DAGO and DPDPE, respectively^25^ (consistent with low KOP surface density in comparison to that of MOP and DOP), it is obvious that NTX acts through more pathways than LY2444296. It is also acting via more abundant pathways, given the higher MOP and DOP surface densities compared to KOP. This broader mechanism of NTX action via several receptors may underly its ability to protect against long-range EtOH-induced reorganization of plasma membrane lipids and proteins, whereas LY2444296, exclusively acting via KOP (Figure S7), is, at the concentrations tested, only affecting short-range interactions, i.e., the immediate KOP surrounding. Second, the pharmacological profile of KOP is complex,^44^ and natively expressed KOP in SH-SY5Y cells may be “less responsive″ to LY2444296 due to alternate splicing or KOP homo- and/or heterodimerization. Further studies in other KOP-expressing cell lines and primary neurons are needed to assess the generalizability of our observation that LY2444296 did not show protective effects against EtOH-induced reorganization of plasma membrane constituents, although it did induce local changes in the immediate, KOP-surrounding Chol/SM cluster populations. Finally, NTX and LY2444296 differ significantly in their chemical structures and biophysical properties, which may influence their interactions with the plasma membrane. NTX is a rigid, heteropentacyclic molecule with a fused tetracyclic ring system and a cyclopropylmethyl group, whereas LY2444296 is a more flexible tetracyclic molecule with three aromatic rings and one heterocyclic ring linked by short aliphatic chains. NTX is also less lipophilic, as reflected by its partition coefficient Log P_NTX_ = 1.9 (n-octanol/buffer, pH 7.4, 37 °C), compared to LY2444296, log*P*_LY2444296_ = 4.4.^45^ Additionally, NTX has a higher topological polar surface area (TPSA), TPSA_NTX_ = 70 Å^2^, compared to TPSA_LY2444296_ = 55.6 Å^2^,^45^ suggesting lower capacity to passively diffuse through the plasma membrane compared to LY2444296. Due to these structural and physicochemical differences, NTX may modulate the dynamic lateral organization of the plasma membrane more effectively than LY2444296, which can more effectively translocate across it.

In line with the notion that SH-SY5Y cells endogenously expresses more MOP than KOP,^27^ and the fact that opioids-bound MOP suppresses the activity of voltage-dependent Ca^2+^ ion channel via binding of G_βγ_ subunits that are released when MOP is activated,^46,47^ we could potentially also explain why statistically significantly elevated intercellular Ca^2+^ levels were observed in SH-SY5Y cells under NTX treatment (Figure 5D); an antagonist would regulate the Ca^2+^ channel in the opposite direction, increasing the basal activity of the voltage-dependent Ca^2+^ channel.

In summary, our data suggest that EtOH enhances Ca^2+^ activity by reorganizing nanodomains in the plasma membrane. This aligns with *in vitro* studies indicating that the lipid environment influences Ca^2+^ transporter activity.^48^ The protective effects of NTX appear to primarily stem from its direct binding to opioid receptors, as our data using the optical enantiomer (+)-NTX suggest, whereas its direct effect on the modulation of plasma membrane lipid organization was secondary (Figure 4B). Our recent findings, using both wild-type PC12 cells and PC12 cells stably transformed to express human KOP receptor genetically fused with the enhanced green fluorescent protein (KOP-eGFP), have shown that NTX counteracts EtOH-induced increase in plasma membrane fluidity and Ca^2+^ influx, supporting the idea that NTX can act both through opioid receptor-mediated and direct effects on the plasma membrane.^49^ Building on this, we further reason that EtOH may induce ER stress and/or disrupt Ca^2+^ homeostasis,^50^ thereby affecting cellular responses to depolarization. This may also explain the unexpected observation that both NTX and LY2444296 further increased intracellular Ca^2+^ levels and our observation that EtOH enhanced depolarization-induced Ca^2+^ influx in a dose-dependent manner, an effect that NTX, but not LY2444296, could block.

## Conclusions and Perspectives

Data obtained here using three complementary techniques, qSMLM, FCS, and functional Ca^2+^ imaging, indicate a profound protective effect of the opioid receptor antagonist NTX on EtOH-induced rearrangement of Chol/SM-enriched domains in SH-SY5Y cells natively expressing opioid receptors. In contrast, the KOP-specific antagonist LY2444296, at the concentration and treatment time tested, did not show such a protective effect. Our data reveal an important effect of NTX on the dynamic lateral organization of proteins and lipids in the plasma membrane that was hitherto unknown. In addition to blocking the orthosteric activity of opioid receptors (canonical function), NTX influences the dynamic lateral organization of lipids in the plasma membrane, affecting physiological outcomes through mechanisms that may not only be opioid receptor-mediated.

These findings introduce a novel dimension to the pharmacological profile of NTX, its ability to preserve the integrity and dynamic lateral organization of Chol/SM harboring domains in the plasma membrane. The concept of targeting plasma membrane organization as a therapeutic strategy is gaining traction, and certain drugs have been shown to either stabilize or disrupt lipid rafts, leading to modulation of signaling pathways implicated in various diseases.^51^ In the context of AUD, EtOH effects on the allostasis of lipids in patients with AUD who do not have liver dysfunction are long known, and EtOH effect on lowering plasma Chol levels during drinking is well established.^52^ Also, opposite changes in plasma Chol levels in abstinent individuals^53^ and the capacity of NTX to blunt Chol rebound in abstinence^54^ have been observed. It may be possible that NTX facilitates Chol sequestration into the plasma membrane of cells, for example, Chol incorporation into the plasma membrane of red blood cells could be one possible mechanism through which NTX reduces Chol levels in blood plasma.^54^ NTX capacity to protect plasma membrane integrity and protect against EtOH-induced changes in the dynamic lateral organization of proteins and lipids in the plasma membrane could therefore be particularly beneficial. By stabilizing the dynamic lateral organization of the plasma membrane, NTX may help plasma membrane receptors maintain proper localization and function, potentially reducing the reinforcing effects of alcohol and aiding in the prevention of relapse. Individual variability in lipid status may also underlie individual responses to NTX pharmacotherapy. This opens new avenues for the development of novel, individually tailored treatments for the prevention of relapses in AUD that target plasma membrane organization. Such compounds could serve as adjunct therapies to existing pharmacological and behavioral interventions. Future research should focus on identifying and characterizing such compounds as well as elucidating the precise mechanisms by which EtOH and NTX exert their plasma membrane-destabilizing/stabilizing effects.

## Supplementary Material



## Data Availability

The original data and materials presented in this study are available upon a request.

## Notes

File contains additional experimental details, materials and methods, six supporting tables, and seven supporting figures (PDF)
