# Supplementary material for: Effects of Ethanol and Opioid Receptor Antagonists Naltrexone and LY2444296 on the Organization of Cholesterol- and Sphingomyelin-Enriched Plasma Membrane Domains
Source: ACS Chem Neurosci. 2025 Sep 26;16(20):4133–46. doi: 10.1021/acschemneuro.5c00596 (PMC12532191; doi:10.1021/acschemneuro.5c00596)
Supplement: Supplementary file 1 [file cn5c00596_si_001.pdf]

Supporting Information:

Effects of Ethanol and Opioid Receptor Antagonists, Naltrexone and LY2444296, on the organization of cholesterol-enriched membrane domains

Sho Oasa[1‡], *Wai H. Mak*[2‡], *Adam L. Maddox*[2], *Carinna Lima*[2], *Andras Saftics*[2], *Lars Terenius*[1], *Tijana Jovanović-Talisman*[2]* *Vladana Vukojević*[1]*

[1]Center of Molecular Medicine, Department of Clinical Neuroscience, Karolinska Institutet, Stockholm 17176, Sweden

[2]Department of Cancer Biology & Molecular Medicine, Beckman Research Institute, City of Hope Comprehensive Cancer Center, Duarte, CA 91010, USA

‡These authors contributed equally

*Corresponding authors: Tijana Jovanović-Talisman, ttalisman@coh.org; Vladana Vukojević, vladana.vukojevic@ki.se

Supplementary Table TS1. Analysis of SMLM data for EtOH treatments (concentration and time dependence)

		**No. cover slips**	**No. cells**	**No. ROIs**	**Medi an**	**Mean ± SEM**	***p-values$_{split}$***
Detected density *(molecules/μm^2)*	Steady State	3	25	83	65.9	72 ± 5	0.5
	10 mM EtOH 1h	3	11	39	67.4	91 ± 8	0.5
	20mM EtOH 1h	3	12	42	89.4	93 ± 6	0.5
	40mM EtOH 1 h	3	12	44	92.9	98 ± 6	0.5
	EtOH 20mM 10 min	3	11	40	96.6	102 ± 7	0.6
	EtOH 20mM 20 min	3	13	49	93.1	84 ± 5	0.6
**Cluster radius** (nm)	Steady State	3	25	83	28.4	27.9 ± 0.7	0.5
	10 mM EtOH 1h	3	11	39	30.5	31.8 ± 1.3	0.4
	20mM EtOH 1h	3	12	42	34.4	34.4 ±1.1	0.5
	40mM EtOH 1 h	3	12	44	30.9	31.7 ± 1.2	0.5
	EtOH 20mM 10 min	3	11	40	30.5	31.4 ± 1.4	0.6
	EtOH 20mM 20 min	3	13	49	24.8	25.8 ± 0.9	0.6
Cluster population *(*molecules/ c*luster)*	Steady State	3	25	83	13.8	17 ± 1	0.4
	10 mM EtOH 1h	3	11	39	15.4	22 ± 3	0.5
	20mM EtOH 1h	3	12	42	22.1	28 ± 3	0.5
	40mM EtOH 1 h	3	12	44	20.0	26 ± 3	0.5
	EtOH 20mM 10 min	3	11	40	18.3	23 ± 3	0.6
	EtOH 20mM 20 min	3	13	49	13.3	15 ± 2	0.5
***Fraction clustered** (%)*	Steady State	3	25	83	36.1	35 ± 2	0.5
	10 mM EtOH 1h	3	11	39	41.0	41 ± 3	0.4
	20mM EtOH 1h	3	12	42	47.9	47 ± 2	0.5
	40mM EtOH 1 h	3	12	44	37.3	39 ± 2	0.4
	EtOH 20mM 10 min	3	11	40	46.6	44 ± 3	0.5
	EtOH 20mM 20 min	3	13	49	26.6	28 ± 2	0.5
**Cl*uster* density** *(clusters/μm^2)*	Steady State	3	25	83	8.1	9 ± 1	0.5
	10 mM EtOH 1h	3	11	39	8.6	12 ± 1	0.4
	20mM EtOH 1h	3	12	42	12.9	14 ± 1	0.5
	40mM EtOH 1 h	3	12	44	11.7	13 ± 1	0.5
	EtOH 20mM 10 min	3	11	40	12.6	16 ± 2	0.5
	EtOH 20mM 20 min	3	13	49	7.9	9 ± 1	0.4

Detected density = the number of detected OlyA-AF647 molecules per unit surface area (molecules/μm^2);

Cluster radius = correlation length (nm);

Cluster population = the number of detected OlyA-AF647 molecules per cluster (molecules/cluster);

Fraction clustered = fraction of OlyA-AF647 molecules in clusters;

Cluster density = the number of detected clusters per unit surface area (number of clusters/μm^2).

Supplementary Table TS2. Summary of *p*-values for EtOH treatments (concentration and time dependence)

	Detected density	Cluster radius	Cluster populat ion	Fraction clustered	Cluster density
Steady State vs. 10 mM EtOH 1h	0.062	0.011	0.148	0.037	0.057
Steady State vs. 20mM EtOH 1h	0.006	<0.0001	0.003	<0.0001	0.001
Steady State vs. 40mM EtOH 1 h	0.002	0.006	0.008	0.131	0.023
10mM EtOH 1h vs. 20mM EtOH 1h	0.805	0.133	0.148	0.092	0.285
10mM EtOH 1h vs. 40mM EtOH 1 h	0.483	0.940	0.269	0.473	0.710
20mM EtOH 1h vs. 40mM EtOH 1 h	0.564	0.089	0.727	0.009	0.520
Steady State vs. 20mM EtOH 10 min	0.002	0.025	0.067	0.005	0.001
Steady State vs. 20mM EtOH 20 min	0.085	0.069	0.335	0.022	0.917
Steady State vs. 20mM EtOH 1h	0.006	<0.0001	0.003	<0.0001	0.001
20mM EtOH 10 min vs. 20mM EtOH 20 min	0.050	0.001	0.016	<0.0001	0.001
20mM EtOH 10 min vs. 20mM EtOH 1h	0.378	0.087	0.215	0.466	0.365
20mM EtOH 20 min vs. 20mM EtOH 1h	0.213	<0.0001	0.001	<0.0001	0.001

Supplementary Table TS3. Analysis of SMLM data for naltrexone and LY2444296 treatments

		No. coverslips	No. cells	No. ROIs	Median	Mean ± SEM	p-values$_{split}$
**Detected density** (molecules/μm^2)	NTX	3	12	47	77.1	89 ± 5	0.5
	NTX-EtOH	3	12	45	76.5	94 ± 8	0.5
	LY	3	12	45	73.4	77 ± 5	0.4
	LY-EtOH	3	11	44	132.2	122 ± 8	0.4
**Cluster radius** (nm)	NTX	3	12	47	28.9	29.0 ± 0.9	0.4
	NTX-EtOH	3	12	45	29.6	29.3 ± 0.9	0.5
	LY	3	12	44	26.9	28.1 ± 0.9	0.6
	LY-EtOH	3	11	44	31.4	32.9 ± 1.4	0.4
**Cluster population** (molecules/ cluster)	NTX	3	12	47	11.1	13 ± 2	0.5
	NTX-EtOH	3	12	45	13.4	14 ± 1	0.5
	LY	3	12	44	10.3	12 ± 1	0.4
	LY-EtOH	3	11	44	15.4	18 ± 2	0.5
**Fraction Clustered (%)**	NTX	3	12	47	33.2	35 ± 2	0.6
	NTX-EtOH	3	12	45	36.4	37 ± 2	0.5
	LY	3	12	44	31.5	32 ± 2	0.5
	LY-EtOH	3	11	44	44.1	44 ± 2	0.4
**Cluster density** (clusters/μm^2)	NTX	3	12	47	9.9	12 ± 1	0.5
	NTX-EtOH	3	12	45	10.3	14 ± 2	0.5
	LY	3	12	44	9.1	10 ± 1	0.5
	LY-EtOH	3	11	44	17.3	20 ± 2	0.5

Supplementary Table TS4. Summary of p-values for naltrexone and LY2444296 treatments

	**Detected density**	**Cluster radius**	**Cluster population**	**Fraction clustered**	**Cluster density**
Steady State vs. EtOH	0.002	0.006	0.008	0.131	0.023
Steady State vs. NTX	0.023	0.338	0.065	0.896	0.038
Steady State vs. NTX-EtOH	0.024	0.221	0.151	0.332	0.009
EtOH vs. NTX	0.275	0.064	0.000	0.176	0.561
EtOH vs. NTX-EtOH	0.681	0.104	0.001	0.551	0.523
NTX vs. NTX-EtOH	0.614	0.797	0.676	0.416	0.230
Steady State vs. LY	0.460	0.856	0.011	0.376	0.339
Steady State vs. LY-EtOH	<0.0001	0.002	0.615	0.002	<0.0001
EtOH vs. LY	0.011	0.015	<0.0001	0.027	0.141
EtOH vs. LY-EtOH	0.025	0.494	0.024	0.111	0.003
LY vs. LY-EtOH	<0.0001	0.004	0.007	0.000	<0.0001

Supplementary Table TS5. Summary of *p*-values for FCS data. Diffusion coefficient (DC) and Counts per molecule (CPM) related to Figure 3 in the main text.

Data 1	Data 2	DC	Density
Untreated	40 mM EtOH	<0.0001	0.99
Untreated	200 nM NTX	<0.0001	0.0002
Untreated	200 nM NTX+40 mM EtOH	<0.0001	<0.0001
Untreated	100 nM LY	0.0003	0.99
Untreated	100 nM LY+40 mM EtOH	<0.0001	0.99
40 mM EtOH	200 nM NTX	0.001	<0.0001
40 mM EtOH	200 nM NTX+40 mM EtOH	0.004	<0.0001
40 mM EtOH	100 nM LY	0.02	1.0
40 mM EtOH	100 nM LY+40 mM EtOH	0.63	0.99
200 nM NTX	200 nM NTX+40 mM EtOH	0.75	0.83
200 nM NTX	100 nM LY	<0.0001	0.0005
200 nM NTX	100 nM LY+40 mM EtOH	0.017	0.0005
200 nM NTX+40 mM EtOH	100 nM LY+40 mM EtOH	0.034	<0.0001
100 nM LY	200 nM NTX+40 mM EtOH	<0.0001	<0.0001
100 nM LY	100 nM LY+40 mM EtOH	0.046	1.0

Supplementary Table TS6. Summary of *p*-values for Calcium imaging data. Baseline and amplitude of Fura-Red ratio (FRr) related to Figure 4 in the main text.

Data 1	Data 2	Baseline	Amplitude
Untreated	10 mM EtOH	<0.0001	0.323
Untreated	40 mM EtOH	<0.0001	0.027
Untreated	200 nM NTX	<0.0001	0.035
Untreated	200 nM NTX+10 mM EtOH	<0.0001	0.0013
Untreated	200 nM NTX+40 mM EtOH	<0.0001	0.0015
Untreated	100 nM LY	0.0002	0.554
Untreated	100 nM LY+10 mM EtOH	<0.0001	0.867
Untreated	100 nM LY+40 mM EtOH	<0.0001	0.183
10 mM EtOH	40 mM EtOH	0.130	0.462
10 mM EtOH	200 nM NTX	0.137	0.404
10 mM EtOH	200 nM NTX+10 mM EtOH	<0.0001	0.074
10 mM EtOH	200 nM NTX+40 mM EtOH	<0.0001	0.058
10 mM EtOH	100 nM LY	0.0003	0.113
10 mM EtOH	100 nM LY+10 mM EtOH	0.0013	0.153
10 mM EtOH	100 nM LY+40 mM EtOH	0.061	0.858
40 mM EtOH	200 nM NTX	0.0013	0.991
40 mM EtOH	200 nM NTX+10 mM EtOH	<0.0001	0.369
40 mM EtOH	200 nM NTX+40 mM EtOH	<0.0001	0.653
40 mM EtOH	100 nM LY	0.065	0.022
40 mM EtOH	100 nM LY+10 mM EtOH	<0.0001	0.049
40 mM EtOH	100 nM LY+40 mM EtOH	0.0002	0.576
200 nM NTX	200 nM NTX+10 mM EtOH	<0.0001	0.125
200 nM NTX	200 nM NTX+40 mM EtOH	<0.0001	0.112
200 nM NTX	100 nM LY	<0.0001	0.0006
200 nM NTX	100 nM LY+10 mM EtOH	0.0086	0.0087
200 nM NTX	100 nM LY+40 mM EtOH	0.319	0.344
200 nM NTX+10 mM EtOH	200 nM NTX+40 mM EtOH	0.909	0.891
200 nM NTX+10 mM EtOH	100 nM LY	<0.0001	<0.0001
200 nM NTX+10 mM EtOH	100 nM LY+10 mM EtOH	<0.0001	<0.0001
200 nM NTX+10 mM EtOH	100 nM LY+40 mM EtOH	<0.0001	0.023
200 nM NTX+40 mM EtOH	100 nM LY	<0.0001	<0.0001
200 nM NTX+40 mM EtOH	100 nM LY+10 mM EtOH	<0.0001	<0.0001
200 nM NTX+40 mM EtOH	100 nM LY+40 mM EtOH	<0.0001	0.023
100 nM LY	100 nM LY+10 mM EtOH	<0.0001	0.644
100 nM LY	100 nM LY+40 mM EtOH	<0.0001	0.012
100 nM LY+10 mM EtOH	100 nM LY+40 mM EtOH	0.094	0.056

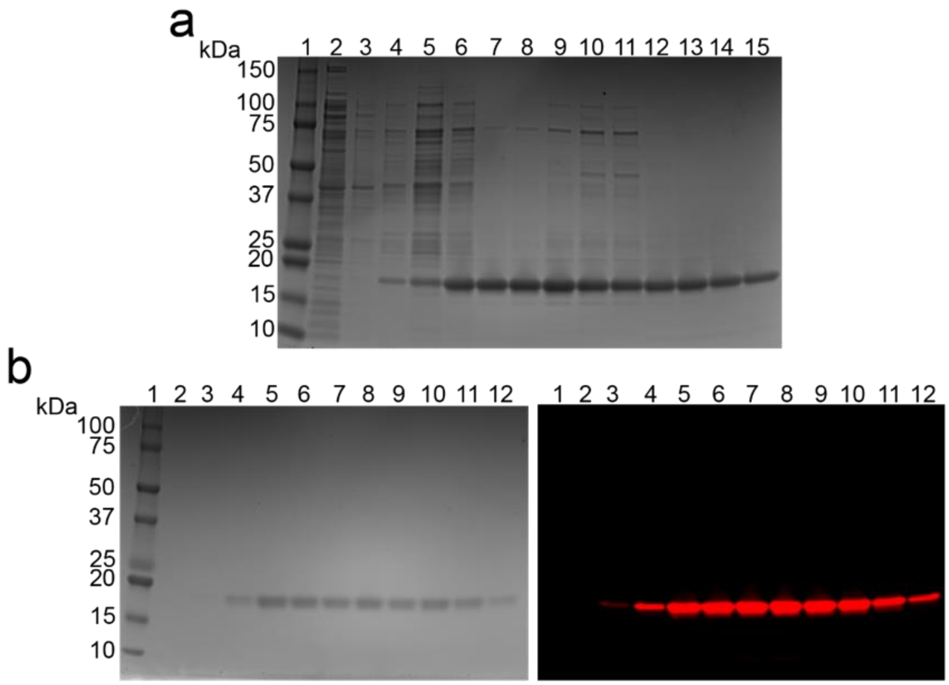

Figure S1: SDS-PAGE gels of purified OlyA and visualization of successful conjugation of AF647 to OlyA. A. OlyA-His_6 was purified using Co-NTA affinity chromatography followed by size exclusion chromatography (SEC). After two-step purification, a band at the expected molecular weight (~17 kDa) was observed. Lanes are: 1, Ladder; 2, flow through; 3, 5 mM imidazole wash; 4, 10 mM imidazole wash; 5, 200 mM imidazole elution (1); 6, 200 mM imidazole elution (2); 7, 200 mM imidazole elution (3); 8, 200 mM imidazole elution (4); 9, 200 mM imidazole elution (5); 10, 200 mM imidazole elution (6); 11, 200 mM imidazole elution (7); 12, SEC fraction C2; 13, SEC fraction C3; 14, SEC fraction C4; 15, SEC fraction C5. Lanes 7,8, and 9 were pooled and loaded onto SEC column. SEC Fractions C3, 4, and 5 were used for labelling. **B.** An aliquot of purified OlyA was conjugated to AF647 through maleimide chemistry via the free cysteine residue. OlyA was purified from free dye in several fractions (lanes 3-12). Labelled, purified protein was assessed using coomassie stain (left image) and a fluorescence imager (right image). Reprinted with permission from[1].

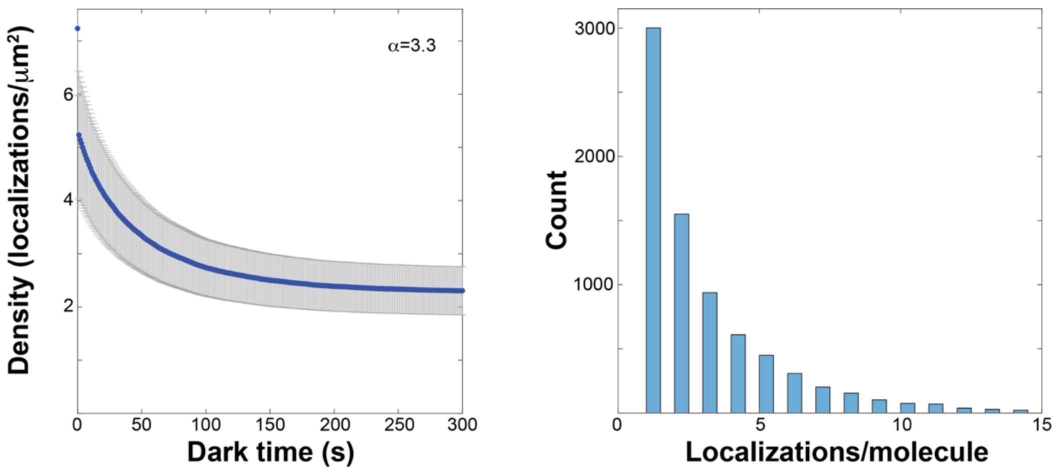

Figure S2. Photophysical properties of OlyA-AF647. Based on 9 ROIs, we determined the average number of localizations per fluorescent probe, $\alpha = 3.3$; and the maximum dark time of 250 s.

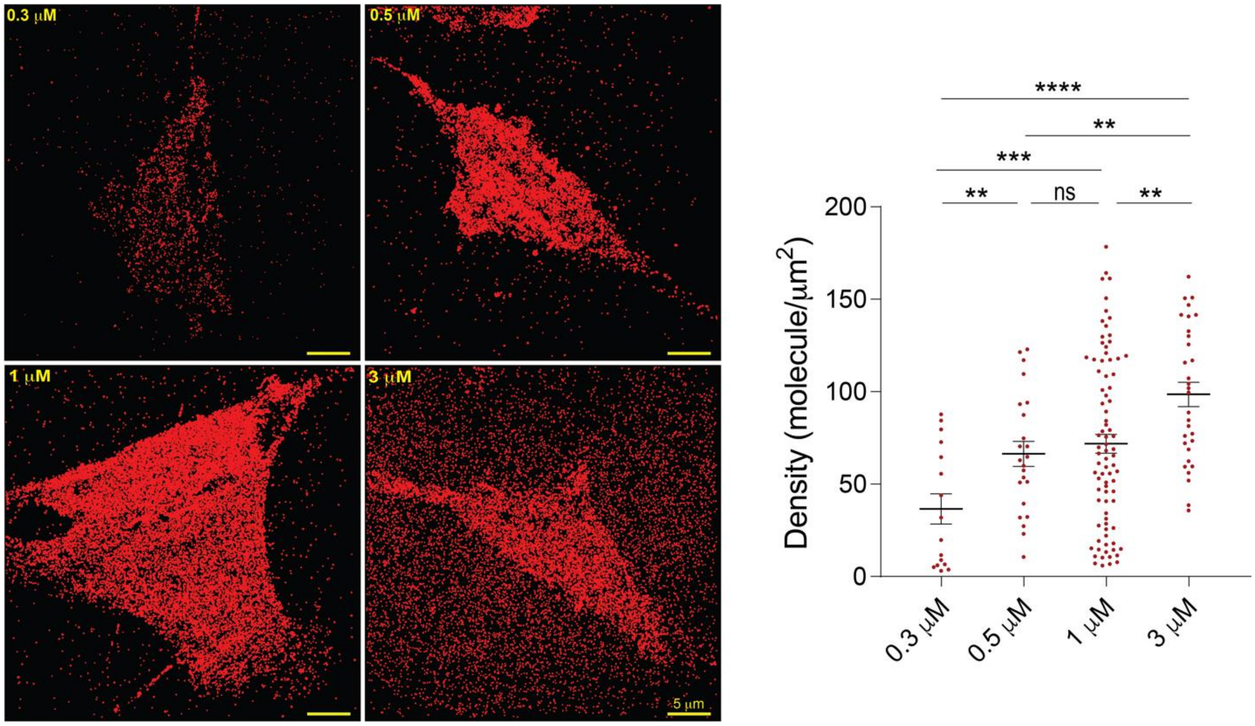

Figure S3. Optimization of the protocol for staining SH-SY5Y cells with OlyA-AF647. SMLM images (left) and quantification of molecular density upon staining with different concentrations of OlyA-AF647 (right).

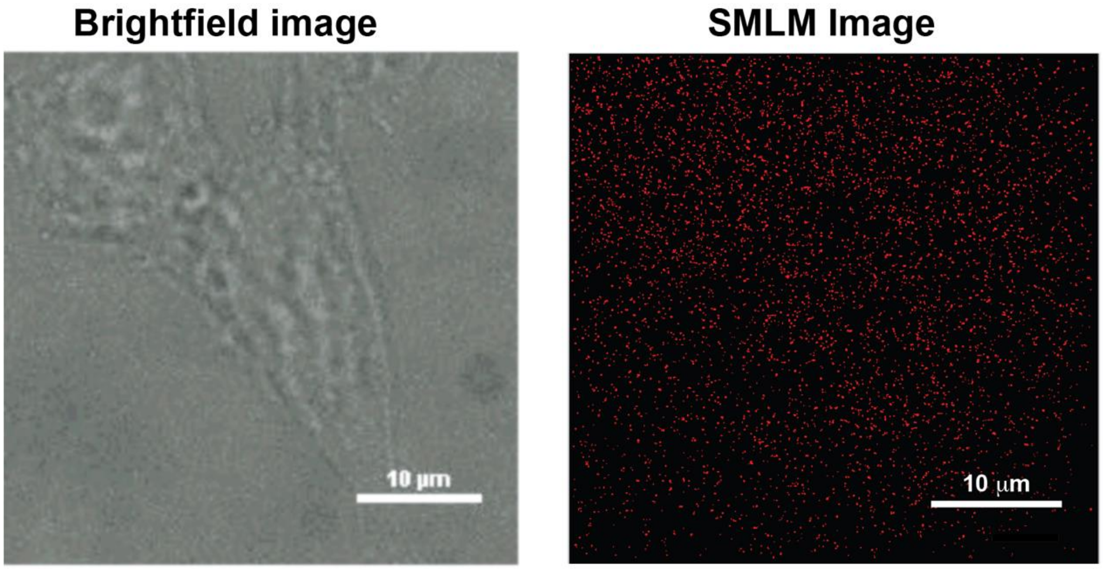

Figure S4. MT-OlyA-AF647 does not bind to the SH-SY-5Y cells. Incubation of SH-SY5Y cells with 1 µM MT-OlyA-AF647 for 15 min, which resulted in most efficient staining with WT-OlyA-AF647 (Fig. S3), resulted in no cell staining. The left panel shows a representative brightfield image and the right panel the corresponding SMLM image.

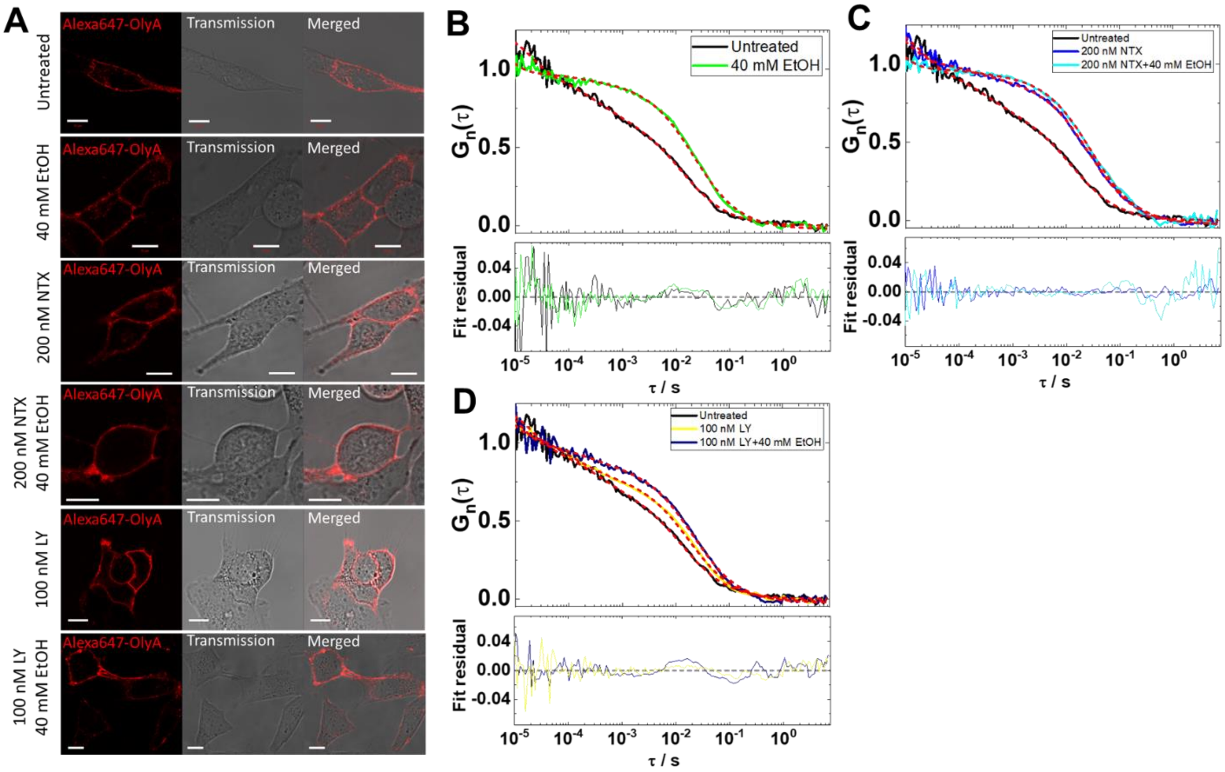

Figure S5. Overall OlyA-AF647 localization in live SH-SY5Y cells is not affected by pharmacological treatment but its dynamics in the plasma membrane is. A. Confocal laser scanning microscopy shows that treatment with EtOH, NTX or LY2444296 (LY), does not alter overall OlyA-AF647 localization in SH-SY-5Y cells. **B-D.** Experimentally derived autocorrelation curves normalized to the same amplitude, $G_n(\tau) = 1$ at $\tau = 10$ μs, (solid lines) fitted using equation (2a; dashed lines) and corresponding fit residuals showing the difference between the experimental and fitted $G_n(\tau)$ values, reveal OlyA-AF647 dynamics under the treatment with 40 mM EtOH (B); 200 nM NTX and pretreatment with 200 nM NTX followed by 40 mM EtOH (C); and 100 nM LY and pretreatment with 100 nM LY followed by 40 mM EtOH (D).

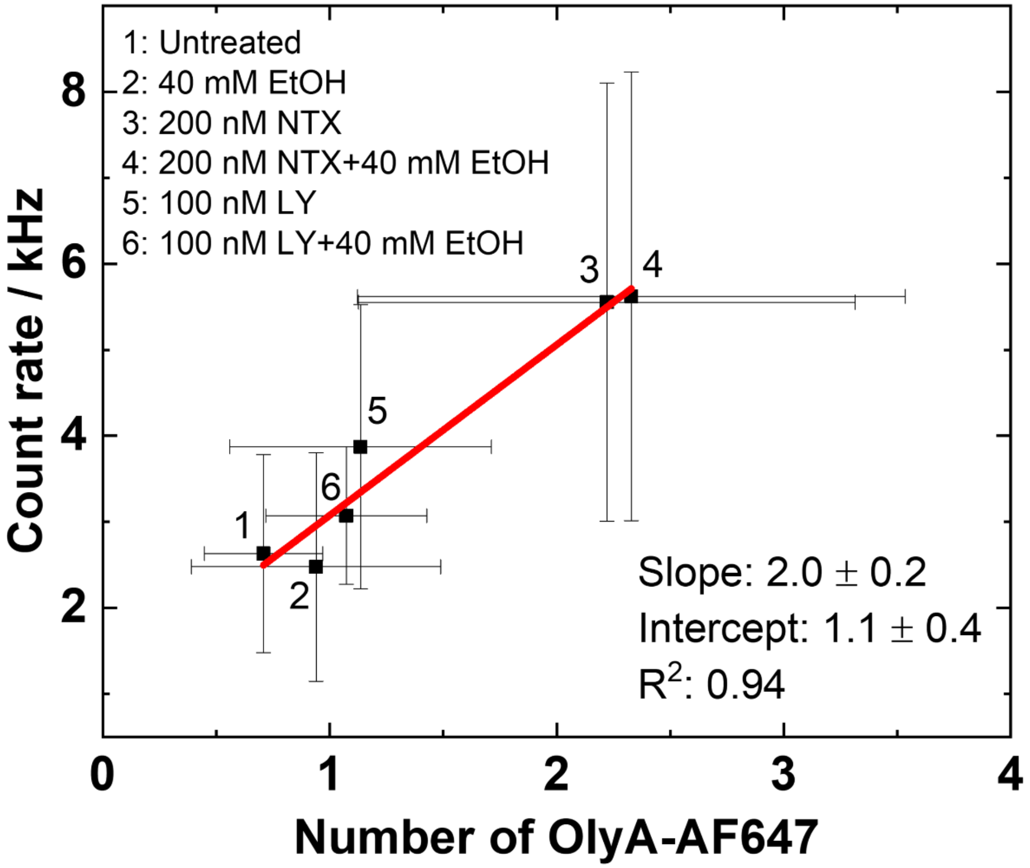

Figure S6. Pharmacological treatments do not change brightness of OlyA-AF647 bound to Chol/SM complexes in the cellular plasma membrane. Average fluorescence intensity linearly increases with the average number of OlyA-AF647 (red line), indicating that OlyA-AF647 brightness is the same, as reflected by the counts *per* second *per* molecule (CPM), corresponding to the slope of linear regression, CPM ≈ 2 kHz/molecule).

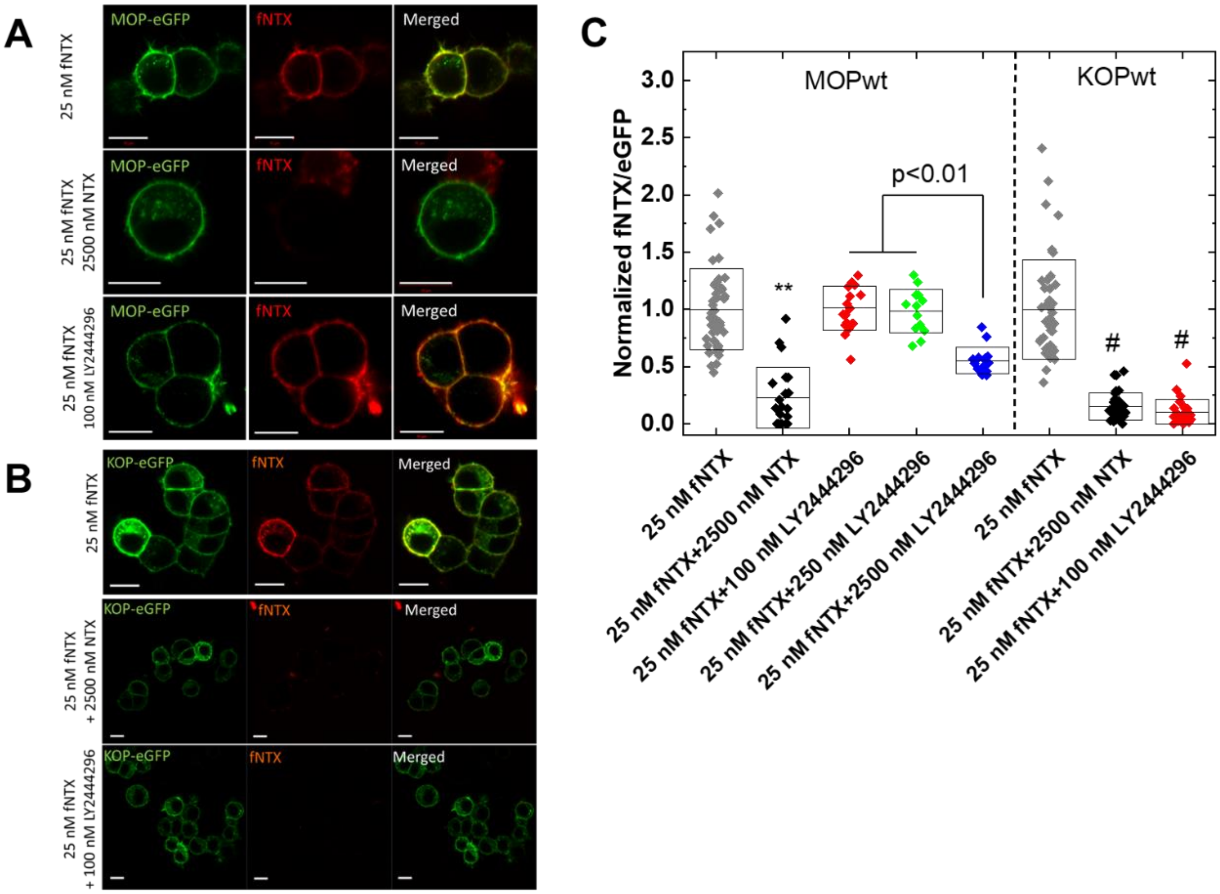

Figure S7. LY2444296 specifically binds to KOP, but not to MOP. (A, B) Confocal Laser Scanning Microscopy (CLSM) images of fluorescently labeled naltrexone (fNTX; red) binding to PC12 cells stably expressing wild type mu-opioid receptor (MOP_{wt}) genetically fused with the enhanced green fluorescent protein (MOP_{wt}-eGFP; A) or KOP_{wt}-eGFP (B). *Top row:* Binding of 25 nM fNTX to MOP_{wt}-eGFP (A) or KOP_{wt}-eGFP (B). *Middle row*: Binding of 25 nM fNTX in the presence unlabeled NTX in a large excess, 2500 nM NTX, to MOP_{wt}-eGFP (A) or KOP_{wt}-eGFP (B). *Bottom row*: Binding of 25 nM fNTX mixed with 100 nM LY2444296 to MOP_{wt}-eGFP (A) or KOP_{wt}-eGFP (B). Scale bar: 10 µm. CLSM images shown in (B) are from [1]. **(C)** The ratio of fluorescence intensities (R) is given as average ± standard deviation. Grey: 25 nM fNTX, Black: 25 nM fNTX+2500 nM NTX, Red: 25 nM fNTX+100 nM LY2444296, Green: 25 nM fNTX+250 nM LY2444296, Blue: 25 nM fNTX+2500 nM LY2444296. Statistical analysis was performed

using one-way ANOVA test with *Turky* test. Asterisks indicate a statistically significant difference with respect to MOP_{wt}-eGFP cells treated with 25 nM fNTX, $p < 0.01$ (**). Hashtag (#) indicates statistically significant difference with respect to KOP_{wt}-eGFP cells treated with 25 nM fNTX, $p < 0.01$.

Supplementary Materials and Methods: Competitive LY2444296 binding assay in PC12 cells

PC12 cells stably transformed to express wild type mu- and kappa-opioid receptors (MOP_{wt} and KOP_{wt}) genetically fused with the enhanced green fluorescent protein (MOP_{wt}-eGFP and KOP_{wt}-eGFP) [2, 3], were maintained in a humidified atmosphere containing 5% CO^2 at 37 °C in RPMI1640 medium (Gibco) supplemented with 10 % horse serum (Gibco), 5 % fetal bovine serum (Gibco) and 1 % penicillin-streptomycin (final conc.: 100 U/mL; Gibco). The cells were seeded in Lab-Tek 8-well chambered cover glass at density of 4.0×10^4 cells/well.

Fluorescently labelled naltrexone (fNTX), naltrexone chemically conjugated with Alexa Fluor 633, was synthesized as previously described [3]. The fNTX in DMSO was diluted to 25 nM working solution with phenol-red free FluoroBriteTM DMEM (Gibco).

The cells were pre-treated with 25 nM fNTX for 30 min, followed by a 30 min treatment using a mixture of 25 nM fNTX and non-labeled antagonists, NTX or LY2444296. For the competitive binding assay in MOP_{wt}-eGFP PC12 cells, 2500 nM NTX and three different concentrations of LY2444296, 100 nM, 250 nM and 2500 nM) were tested. The following concentrations, 2500 nM NTX or 100 nM LY2444296 were tested in KOP_{wt}-eGFP PC12 cells.

The competitive binding assay was performed using the LSM880 confocal microscope system used throughout. The 488 nm and 633 nm lasers were focused using the water immersion microscope objective (C-Apochromat, 40×, 1.2 N.A., Corr). eGFP and fNTX were excited using the 488 nm line of the Ar-laser and the 633 nm HeNe laser, respectively. eGFP fluorescence was recorded in the 500-530 nm wavelength range using the GaAsP detector. fNTX fluorescence was recorded in the 650-700 nm wavelength range using the photomultiplier tube (PMT). The pinhole size was 1 Airy Unit, 34 µm for eGFP and 45 µm for fNTX. To avoid signal crosstalk, the multi-track imaging mode was used.

The ZEN software was used to make line profiles across the plasma membrane, and maximum fluorescence intensity in the line profile reflected fluorescence intensity at the plasma membrane. The fluorescence intensity ratio, R_i was calculated as follows [3]:

$$R_i = \frac{I_{fNTX}}{I_i}$$

where I_{fNTX} is the fluorescence intensity in the red channel, and I_i is the fluorescence intensity in the green channel, reflecting on MOP_{wt}-eGFP or KOP_{wt}-eGFP surface density.

References

(1) Maddox, A. Employing Single Molecule Localization Microscopy to Assess Molecular Features of HER2 in Breast Cancer. Irell and Manella Graduate School of Biological Sceinces, 2022.

(2) Rogacki, M. K.; Golfetto, O.; Tobin, S. J.; Li, T.; Biswas, S.; Jorand, R.; Zhang, H.; Radoi, V.; Ming, Y.; Svenningsson, P.; et al. Dynamic lateral organization of opioid receptors (kappa, muwt and muN40D) in the plasma membrane at the nanoscale level. *Traffic* **2018**. DOI: 10.1111/tra.12582 From NLM.

(3) Oasa, S.; Sezgin, E.; Ma, Y.; Horne, D. A.; Radmilović, M. D.; Jovanović-Talisman, T.; Martin-Fardon, R.; Vukojević, V.; Terenius, L. Naltrexone blocks alcohol-induced effects on kappa-opioid receptors in the plasma membrane. *Translational Psychiatry* **2024**, *14* (1), 477. DOI: 10.1038/s41398-024-03172-8.
